# Removal of Pb(II) from Water by Potassium Permanganate-Modified Areca Fiber Biochar: Performance and Mechanism

**DOI:** 10.3390/molecules31193437

**Published:** 2026-09-27

**Authors:** Pingping Yang, Yunquan Liu, Wanxin Cheng, Yaping Cheng, Wei Zhang

**Affiliations:** 1 Guangxi Key Laboratory of Environmental Pollution Control Theory and Technology, Guilin University of Technology, Guilin 541006, China; 2 University Engineering Research Center of Watershed Protection and Green Development, Guilin University of Technology, Guilin 541006, China; 3 Key Laboratory of Carbon Emission and Pollutant Collaborative Control, Education Department of Guangxi Zhuang Autonomous Region, Guilin University of Technology, Guilin 541006, China; 4 College of Civil Engineering and Architecture, China Three Gorges University, Yichang 443002, China

**Keywords:** potassium permanganate, areca nut-derived biochar, Pb(II) removal, mechanism

## Abstract

Heavy metal contamination, particularly lead (Pb) pollution in aquatic environments, poses serious threats to ecosystems and human health, necessitating the development of efficient and cost-effective adsorbents. In this study, potassium permanganate-modified areca fiber biochar (PAFB) is successfully synthesized for Pb(II) removal from aqueous solutions. Characterization results confirm the successful loading of MnO_2_ nanoparticles and the introduction of abundant –OH, –COOH, and Mn–OH active sites onto the biochar surface. Batch adsorption experiments reveal that Pb(II) removal by PAFB is highly pH- and temperature-dependent, with optimal performance at pH = 6.0 and 25 °C. The adsorption process follows the pseudo-second-order kinetic model *(R*^2^ = 0.9997) and the Freundlich isotherm model (*R*^2^ = 0.9363), indicating chemisorption-dominated adsorption on heterogeneous surfaces. Thermodynamic analysis confirmed the spontaneous and endothermic nature of the adsorption process. The maximum adsorption capacity reaches 45.62 mg/g at 25 °C, comparable to or higher than that of many similar biochar-based adsorbents. Mechanistic analysis revealed that Pb(II) removal by PAFB proceeds via a synergistic electrostatic attraction–surface complexation mechanism, wherein Pb(II) forms inner-sphere complexes with –OH, –COOH, and ≡Mn–OH groups, as evidenced by FTIR and XPS analyses. The Pb4f spectrum exclusively displayed Pb(II) complexation peaks without detectable Pb^0^ precipitates, confirming the immobilization of Pb(II) as amorphous surface complexes. Moreover, PAFB maintains an 87.00% removal efficiency after four regeneration cycles, demonstrating excellent reusability. This study provides an environmentally friendly adsorbent for Pb(II) removal, showing promising potential for heavy-metal-contaminated wastewater treatment.

## 1. Introduction

The rapid pace of industrialization and urbanization has led to the widespread discharge of heavy-metal-contaminated wastewater into aquatic ecosystems, posing severe risks to both environmental sustainability and public health. Compared with other toxic heavy metals such as Cd(II) and Co(II), lead (Pb) is recognized as one of the most toxic, prevalent, and persistent pollutants due to its pronounced bioaccumulation, non-biodegradability, and potent toxicity toward multiple organ systems, even at trace concentrations (<5 µg/dL) [1,2,3]. Pb mainly enters the water environment through industrial wastewater generated from lead–acid battery manufacturing, mining and smelting, electroplating, and metal surface treatment [2,4]. Pb ions in water exist in three different forms: particulate lead bound to sediments, dissolved ionic lead (Pb(II)), and relatively rare organ lead compounds [4]. Pb(II) is listed by the World Health Organization (WHO) and the United States Environmental Protection Agency (USEPA) as a priority heavy-metal pollutant. The maximum contaminant level for Pb(II) in drinking water has been strictly set at 0.015 mg/L by the USEPA and 0.01 mg/L by the WHO [1]. Moreover, prolonged exposure to Pb(II) can cause severe neurological disorders, renal dysfunction, reproductive system damage, and developmental abnormalities [5,6]. More importantly, Pb(II) exerts particularly detrimental effects on the intellectual development of children [7]. Therefore, the effective removal of Pb(II) from water sources is an urgent environmental priority.

A variety of techniques have been developed for Pb(II) removal from contaminated water, including chemical precipitation [8], ion exchange [9], membrane filtration [10], electrochemical treatment [11], and adsorption [12,13]. Among these, adsorption has emerged as one of the most promising approaches owing to its distinct advantages of high efficiency, operational simplicity, cost-effectiveness, and minimal secondary pollution [14,15,16,17]. The performance of adsorption processes largely depends on the physicochemical properties of the adsorbent materials, such as specific surface area and pore structure, the presence of surface functional groups, and chemical stability [12,14,18,19]. Consequently, the rational design and development of low-cost, high-performance adsorbents have become a central focus in this field.

In recent years, various adsorbents, including activated carbon [20], graphene oxide [19], metal–organic frameworks [13], layered double hydroxides [21], and agricultural waste-derived biochars [15], have been extensively explored for heavy metal removal. Among these, biochar has attracted particular attention due to its renewable feedstock sources, low production cost, and favorable surface chemistry [22,23]. However, pristine biochar typically exhibits limited adsorption capacity for heavy metal cations because of its relatively low surface functionality and insufficient active sites. At 25 °C, pH = 5.0, dosage = 0.1 g, *V* = 80 mL, and a contact time of 24 h, the Langmuir-fitted maximum adsorption capacity of coconut shell biochars for Pb(II) is 32.31 mg/g [23]. Under similar conditions, the Langmuir-fitted maximum adsorption capacity of Mn-modified bamboo-derived biochar for Pb(II) is 37.24 mg/g [24]. Furthermore, pine wood biochars pyrolyzed at 400 °C and 700 °C exhibited Pb(II) adsorption capacities of 45.43 mg/g and 40.71 mg/g, respectively, at pH = 5.5, with a reaction time of 2 d [18]. To overcome these limitations, chemical modification strategies, such as acid/alkali treatment [4,25], metal oxide loading [18,24], and surface functionalization [26,27], have been proposed. These methods have been developed to enhance the adsorption performance of biochar-based materials. Among these modification approaches, potassium permanganate (KMnO_4_) has garnered increasing interest because it can simultaneously introduce manganese oxides and oxygen-containing functional groups onto biochar surfaces, creating abundant active sites for heavy metal complexation [28,29,30].

Furthermore, the selection of appropriate biomass feedstocks for biochar production is equally critical for achieving sustainable and economically viable wastewater treatment. Areca fiber (AF) is a natural lignocellulosic material derived from the husks of areca nuts and is abundantly available in tropical and subtropical regions as an agricultural byproduct [31,32]. It possesses inherent advantages such as low cost, renewability, high mechanical strength, and a favorable fibrous structure [33,34]. However, the potential of areca fiber as a precursor for biochar-based adsorbents has rarely been explored. To date, the majority of relevant studies have focused on the utilization of areca fiber or its biochar for the adsorption of dyes [35,36,37] and the enhancement of interfacial bonding strength [38,39,40], with limited attention paid to systematic modification strategies to enhance Pb(II) adsorption performance. In addition, there is still a lack of objective understanding of the potential mechanisms of removing Pb(II) using KMnO_4_-modified betel nut fiber biochar.

Hence, this study aims to synthesize potassium permanganate-modified areca fiber biochar (PAFB) via the KMnO_4_ modification of areca fiber-derived biochar. The Pb (II) adsorption performance of PAFB is systematically investigated under different operational conditions, including variations in pH, solid–liquid ratio, initial Pb(II) concentration, contact time, temperature, and coexisting ions. The physicochemical properties of PAFB are characterized using various analytical techniques, including BET, XRD, FTIR, XPS, and Zeta potential measurements. The underlying adsorption mechanisms are elucidated by integrating characterization results with macroscopic adsorption data and kinetic, isotherm, and thermodynamic analyses. The findings of this work are expected to provide both theoretical insights and practical guidance for the development of high-performance, cost-effective biochar-based adsorbents derived from agricultural wastes for heavy metal removal from aqueous environments.

## 2. Results and Discussion

### 2.1. Material Characterization

#### 2.1.1. SEM-EDS

Figure 1 shows the surface morphology (SEM) and elemental composition (EDS) of areca fiber biochar (AFB) and PAFB before and after Pb(II) adsorption. At a magnification of 1000 times, the surface of AFB has a scale-like structure (Figure 1a). Upon further magnification, it is found that the size of a single scale is about 20–30 μm (Figure 1b). Particles with a size of about 8–10 μm are distributed within the scale-like structure (Figure 1c). Meanwhile, AFB exhibited a well-defined tubular channel structure. This feature is primarily attributed to the carbon skeleton characteristics preserved from the natural anisotropic tissue of areca fiber during the pyrolysis process [37]. After KMnO_4_ modification, the surface morphology of PAFB underwent significant changes compared with those for AFB (Figure 1e–g). Specifically, the scale-like structure raised on the surface of PAFB is destroyed, which makes the tubular river structure more prominent (Figure 1e,f). In addition, the granular particles, approximately 8 µm in size, are observed to be uniformly embedded within the tubular channels (Figure 1g). Upon closer inspection, these granular particles are agglomerates of finer microstructural grains measuring 2–4 µm. After Pb(II) adsorption, the protruding scale-like morphology on the surface of PAFB (with a radius of approximately 100 µm) is further diminished, whereas the tubular channels remain largely unchanged (Figure 1i–k). However, compared with the appearance before adsorption, the tubular channels of PAFB-Pb are covered with a substantial amount of granular substances (Figure 1j,k). These granular deposits are speculated to be lead-containing compounds formed during the adsorption process [1].

For EDS analysis, three independent areas are randomly selected for each sample at 5000 magnification. Within each area, three points are analyzed, and each sample has a total of nine points. The points are selected to avoid edges, cracks, pores, and obvious impurities and to cover both bright non-rich and dark carbon-rich domains. The element contents are reported as average values.

Therefore, EDS analysis revealed that AFB is primarily composed of carbon (C), nitrogen (N), and oxygen (O), with approximate contents of 68.40%, 6.90%, and 24.70%, respectively (Figure 1d). Compared with AFB, the EDS results for PAFB reveal the emergence of Mn (0.30%) and K (0.40%) (Figure 1h). Although the Mn and K contents are relatively low, this observation nonetheless confirms the successful immobilization of KMnO_4_ onto the AFB surface. Meanwhile, the N content decreases slightly from 6.90% to 4.30%. This reduction is likely attributable to the partial oxidative degradation of nitrogen-containing active sites (pyrrolic-N and amine groups, etc.) within the biochar matrix induced by the strong oxidizing nature of KMnO_4_ [41,42]. After Pb(II) adsorption, the O content decreases from 24.10% to 20.20%, and the Pb content is 0.20% (Figure 1l). Meanwhile, compared with PAFB, the C and N contents of PAFB-Pb increased by 0.80% and 3.10%, respectively. This suggests that oxygen-containing functional groups (including hydroxyl, carboxyl, and Mn–O, etc.) likely served as the primary coordination sites, whose availability is partially masked by the adsorbed Pb species [42]. The increase in the N content may be due to the co-adsorption of nitrate ions (${NO}_{3}^{-}$) from Pb(NO_3_)_2_.

#### 2.1.2. BET

Figure 2 shows the N_2_ adsorption–desorption measurements of AF, AFB, and PAFB and shows that the specific surface area, pore volume, and pore diameter of the three groups of samples have an obvious gradual evolution trend. The raw AF has an *S*_BET_ of 7.08 m^2^/g, a pore volume of 0.15 cm^3^/g, and an average pore size of 8.41 nm. This indicates that the pristine biomass material features a dense structure, with relatively underdeveloped pores. After high-temperature pyrolysis activation, compared with AF, the AFB exhibits a remarkable increase in *S*_BET_ to 20.35 m^2^/g (an increase of 187%). Its pore volume doubles to 0.30 cm^3^/g, while the average pore size decreases to 5.80 nm. This change is attributed to the pyrolysis of cellulose, hemicellulose, and lignin, releasing volatile matter and generating abundant newly formed mesopores within the carbon skeleton [31]. Meanwhile, carbon components undergo polycondensation–aromatization, and some macropores contract and collapse, thus reducing the average pore size of the material [34]. After oxidation treatment with KMnO_4_, compared to the results for PAF, the *S*_BET_ of PAFB is reduced to 11.06 m^2^/g (a decrease of 45.6%). The pore volume is sharply decreased to 0.018 cm^3^/g (a decrease of 94.0%), whereas the average pore size is increased to 6.62 nm. This is mainly associated with in-pore deposition and pore blocking by MnO_2_ nanoparticles [42]. Meanwhile, according to the IUPAC classification [43], all three groups of samples show typical Type IV isotherms, consistent with the characteristic adsorption behavior of mesoporous materials (2–50 nm) (Supplementary Material, Figure S1). Specifically, in the low-pressure region (*P*/*P*_0_ < 0.2), the adsorbed volume increases slowly, indicating the presence of a small amount of micropore adsorption. In the medium-pressure region (*P*/*P*_0_ = 0.4–0.8), the adsorption capacity rises gradually. In the high-pressure region (*P*/*P*_0_ > 0.8), the adsorbed volume increases sharply. This phenomenon is generally attributed to capillary condensation within mesopores [44]. Additionally, all samples have H3 hysteresis loops, and the desorption curve is higher than the adsorption curve. These characteristics generally correspond to those of mesopores formed by slit-shaped pores or the stacking of sheet-like particles [44]. Notably, the hysteresis loop of AFB becomes narrower compared with that of AF. This indicates that during pyrolysis and carbonization, abundant newly formed mesopores are generated in the material, and shrinkage of the carbon skeleton occurs, resulting in reduced pore size, as well as increased specific surface area and pore volume. This finding is consistent with the data presented in Figure 2. After modification, the pore volume decreases sharply owing to the blockage of pore channels by MnO_2_ nanoparticles. This demonstrates that the variation in pore structure is dominated by chemical modification rather than resulting from the pyrolysis process.

#### 2.1.3. XRD

Figure 3 shows the XRD patterns of AFB, PAFB, and PAFB-Pb. A broad diffuse diffraction peak is observed for AFB at 2*θ* = 15–30°, which is assigned to the characteristic peak of amorphous carbon. This indicates that the pyrolyzed areca-fiber biochar is mainly composed of an amorphous carbon structure [31]. After modification with KMnO_4_, the new diffraction peaks are observed for PAFB at 2*θ* = 19.14°, 21.89°, 27.57°, and 55.48°, which match the characteristic crystal planes of (200), (101), (201), and (212) of MnO_2_ (JCPDS No. 43-1455) [45]. The mineral phase is identified as ramsdellite. It is confirmed that MnO_2_ nanoparticles have been successfully loaded onto the surface of AFB. After Pb(II) adsorption, the intensities of the characteristic diffraction peaks of MnO_2_ in the XRD patterns of PAFB-Pb are remarkably weakened, and some peaks at certain positions (e.g., at 19.14°) even disappear. Meanwhile, no new diffraction peaks corresponding to Pb or Pb-containing compounds are detected. It is suggested that Pb(II) is mainly captured in an amorphous form on the surface of PAFB, rather than forming crystalline Pb precipitates.

#### 2.1.4. FTIR

The FTIR spectra of AFB, PAFB, and PAFB-Pb are shown in Figure 4. The broad band of AFB at 3422 cm^−1^ is assigned to the stretching vibration of the hydroxyl groups (–OH) [46]. The peaks at 2925 cm^−1^ and 2857 cm^−1^ are attributed to the asymmetric and symmetric C–H stretching vibrations of the methyl and methylene groups in lipids and proteins, respectively [47]. The absorption band at 1703 cm^−1^ corresponds to the stretching vibration of carboxyl groups (C=O), which is the characteristic peak of organic acids in AFB [41]. The peak at 1616 cm^−1^ is ascribed to the C=O stretching vibration of the amide I band, the peak at 1515 cm^−1^ is assigned to the N–H bending vibration of the amide II band, and the absorption band at 1384 cm^−1^ is attributed to the C–H bending vibration [41]. This indicates that abundant oxygen-containing functional groups are present on the surface of pyrolyzed AFB.

After modification with KMnO_4_, the absorption intensities of the –OH band near 3422 cm^−1^ and the C=O stretching-vibration peak of the amide I band at 1616 cm^−1^ are relatively enhanced. Meanwhile, a new C–O stretching-vibration band is observed at 1045 cm^−1^, demonstrating that abundant hydroxyl and carboxyl groups are introduced onto biochar surfaces by KMnO_4_ oxidation treatment. Moreover, metal–π coordination interactions are formed between Mn-containing oxides and surface hydroxyl groups, which makes further improvement in adsorption performance possible [41]. Obvious changes are observed in the FTIR spectra of PAFB-Pb after Pb(II) adsorption. Specifically, the intensities of the –OH absorption band at 3425 cm^−1^ and the C=O stretching–vibration band at 1631 cm^−1^ are remarkably reduced. Meanwhile, both the C–O–C band at 1159 cm^−1^ and the C–O band at 1045 cm^−1^ are weakened to varying degrees. These variations indicate that oxygen-containing functional groups, such as hydroxyl and carboxyl groups on PAFB surfaces, are directly involved in the complexation of Pb(II). Oxygen atoms from these groups are bonded to Pb(II) via coordination bonds, so that the vibration of corresponding chemical bonds is suppressed. In addition, the characteristic band assigned to Mn–O stretching vibration at 608 cm^−1^ shows no obvious shift after adsorption, whereas its intensity is decreased. This further reveals the complexation of Pb(II) by the surface hydroxyl sites (≡Mn–OH) on MnO_2_ [48]. Collectively, FTIR analysis demonstrates that the adsorption mechanism of Pb(II) onto PAFB is closely related to surface oxygen-containing functional groups (–OH and –COOH), as well as Mn–O groups.

#### 2.1.5. XPS

The deconvoluted XPS spectra of PAFB before and after Pb(II) adsorption are peak-fitted, and the results are presented in Figure 5. The C1s spectrum before adsorption is deconvoluted into four characteristic peaks: M–C (282.72 eV, 2.55%), C–C/C=C (284.90 eV, 52.05%), C–O (286.37 eV, 30.08%), and C=O (288.06 eV, 13.14%) (Figure 5a) [49,50]. After Pb(II) adsorption, the C–O peak of PAFB-Pb is shifted toward lower binding energy, and its relative content decreased to 24.52%. The C=O peak is shifted to 288.32 eV, with its relative content remarkably reduced to 10.38%. The M–C peak is positively shifted to 282.97 eV, accompanied by an increase in its relative content to 3.78%. By contrast, the binding-energy position of the C–C/C=C peak remained unchanged, while its relative content increased to 57.58%. The decrease in the relative contents of the C–O and C=O peaks indicates that oxygen atoms from the hydroxyl and carboxyl groups are bonded with Pb(II) via coordination bonds. Their XPS signals are weakened owing to surface coverage or the rearrangement of electron-cloud density. In the N1s spectrum, two characteristic peaks are observed at 400.72 eV (17.65%) and 398.59 eV (38.24%), which are assigned to N–O and C–N bonds, respectively (Figure 5b) [51]. After Pb(II) adsorption, compared with the results for PAFB, the relative contents of both the N–O and C–N bonds are decreased by 5.89%. This suggests that nitrogen-containing functional groups may participate in Pb(II) complexation via the formation of N–Pb coordination bonds. The high-resolution O1s XPS spectrum of PAFB is deconvoluted into three characteristic peaks after peak fitting: 530.18 eV (M–O/M–OH, 29.03%), 532.36 eV (O–C–N, 26.88%), and 533.75 eV (C=O, 22.58%), respectively (Figure 5c) [52]. Among them, the M–O and M–OH peaks confirm the successful loading of MnO_2_ nanoparticles on the biochar surface and the existence of surface hydroxyl sites, which are the key active sites for the surface complexation of Pb(II). Moreover, the O–C–N peak indicates that oxygen in the nitrogen-containing functional groups is involved in the construction of the surface chemical environment. The C=O peak is derived from the intrinsic oxygen-containing functional groups (carboxyl/carbonyl groups) of biochar. These observations confirm the FTIR analysis presented in Section 2.1.4. Two main peaks are observed at 643.09 eV and 652.58 eV in the high-resolution Mn2p spectrum, which are assigned to Mn2p_3/2_ (Mn(II), 44.44%) and Mn2p_1/2_ (Mn(IV), 22.22%), respectively (Figure 5d) [53,54]. This further confirms that the manganese species in PAFB mainly exist in the form of MnO_2_. After Pb(II) adsorption, the Mn(II) peak is shifted toward lower binding energy at 642.61 eV, and its relative content is increased to 44.78%. In contrast, the Mn(IV) peak is shifted toward higher binding energy at 654.13 eV, with its relative content decreased by 7.29%. In addition, two characteristic peaks are observed at 138.15 eV and 143.24 eV in the high-resolution Pb 4f XPS spectrum (Figure 5e), which are assigned to Pb 4f_7/2_ (66.67%) and Pb 4f_5/2_ (33.33%), respectively [14,17]. Notably, no characteristic peaks of Pb^0^ are detected in the spectrum. It is demonstrated that Pb(II) is mainly adsorbed on the surface of PAFB in the form of amorphous surface complexes, rather than being removed via reduction or precipitation pathways. This result is consistent with the observations from XRD (absence of crystalline Pb peaks) and FTIR (decrease in –OH and C=O peak intensities).

### 2.2. Influencing Factors

#### 2.2.1. Effect of pH

The pH of aqueous solution is one of the major factors affecting heavy-metal ion adsorption because pH can alter the surface charge properties of adsorbents and change the speciation of heavy-metal ions in water [55]. Under acidic conditions, the surface of the adsorbent is protonated, and it is difficult for heavy metal ions to approach the adsorbent. When the aqueous solution is alkaline, hydroxyl ions will combine with heavy metal ions in the solution to form precipitates [56]. This may interfere with the actual adsorption process of Pb(II). Therefore, many studies set the pH value range of the solution to 2–7 or 3–11 [10,12,21,25].

The effect of solution pH on Pb(II) adsorption by AFB and PAFB is investigated under the following conditions: initial Pb(II) concentration of 15 mg/L, solution volume of 50 mL, adsorbent dosage of 50 mg, reaction temperature of 25 °C, contact time of 2880 min, and pH ranging from 2 to 7. The results are shown in Figure 6. The Pb(II) removal efficiency (*R*_e_) of AFB increased with the rise in pH value (Figure 6a). The minimum *R*_e_ value of AFB for Pb(II) is 0.91% at pH = 2, while the maximum *R*_e_ value reaches 57.96% at pH = 7. After modification with KMnO_4_, both the *R*_e_ values and equilibrium adsorption capacity (*Q*_e_) of PAFB significantly increased (Figure 6a,b). Specifically, the minimum *R*_e_ and *Q*_e_ values are 28.45% and 4.08 mg/g at pH = 2, respectively. Compared with the results for AFB, the *R*_e_ value is enhanced by 27.54%. The maximum *R*_e_ and *Q*_e_ values reach 96.16% and 13.79 mg/g at pH = 6. When the pH value increased to 7, the *R*_e_ and *Q*_e_ values decreased by 13.27% and 1.9 mg/g, respectively. This trend can be attributed to the distribution of Pb species in the solution, and the charge types on the adsorbent surface are closely dependent on pH values [57,58]. Specifically, Pb(II) may present in the dissolved state species of Pb^2+^, Pb(OH)_2_, ${Pb(OH)}_{3}^{+}$, PbOH^+^, Pb_2_OH^3+^, ${{Pb}_{3}(OH)}_{4}^{2+}$, and ${{Pb}_{4}(OH)}_{4}^{4+}$ at different pH values [59]. In the range of pH = 2.0–6.0, the main forms of Pb^2+^ and ${PbNO}_{3}^{+}$ in the solution are about 90% and 9%, respectively. Meanwhile, the zeta-potential variations of PAFB under the pH range of 3.0–7.0 reveal that PAFB is negatively charged (Supplementary Material, Figure S2). The absolute value of the zeta potential exhibits a monotonically decreasing trend with increasing pH, gradually decreasing from −14.97 mV at pH = 3.0 to −23.95 mV at pH = 6.0. This implies that the negative charge density on the material surface increases substantially. Therefore, the stronger the negative electrification on the material surface, the greater the electrostatic attraction toward Pb^2+^, and the removal efficiency increases accordingly. At pH = 7, the proportion of Pb^2+^ in the solution decreases to 73.95%, and it is converted into PbOH^+^ (17.49%) with a larger hydration radius. The PbOH^+^ scarcely formed any inner-sphere complexes with the surface sites of the material, resulting in the decline in adsorption capacity. In summary, pH = 6 is selected for subsequent investigations on environmental influencing factors.

#### 2.2.2. Effect of *m*/*v*

Figure 7 shows the effect of solid–liquid ratio (*m*/*v*, 0.5–3.0 g/L) on the equilibrium adsorption capacity (*Q*_e_) and removal efficiency (*R*_e_) of PAFB for Pb(II). As the *m*/*v* value increases from 0.5 g/L to 3.0 g/L, the *Q*_e_ values decrease from 9.11 mg/g to 4.67 mg/g, whereas the *R*_e_ values increase significantly from 31.44% to 96.68%. The decreases in *Q*_e_ values are attributed to the reduction in the total amount of Pb(II) corresponding to the per unit mass of the adsorbent with the increase in solid–liquid ratio. Namely, more adsorbent dosage provides abundant active sites, so the amount of Pb(II) adsorbed per unit mass of adsorbent is reduced accordingly [22]. Meanwhile, the agglomeration effect among particles under high adsorbent dosage may also lower the utilization efficiency of surface-active sites. In contrast, the continuous increase in *R*_e_ values indicates that increasing the adsorbent dosage can supply more available active sites for the binding of Pb(II), so that the total removal ratio of Pb(II) in aqueous solution is improved. When the *m*/*v* value reaches 1.0 g/L, the *R*_e_ value is 80.34%. It further increases to 95.97% and tends to be stable as the *m*/*v* value rises to 1.5 g/L. The maximum *R*_e_ value of approximately 96.72% is maintained within the *m*/*v* value range of 2.0–3.0 g/L. Comprehensively considering the adsorption capacity, removal efficiency, and economic cost, 1.0 g/L is selected as the optimal *m*/*v* for subsequent experiments.

#### 2.2.3. Effect of Coexisting Ions

Figure 8 shows the results regarding the effects of coexisting ions (${SO}_{4}^{2-}$, ${HPO}_{4}^{2-}$, Ca^2+^, Mn^2+^, and Mg^2+^, *C*_0_ = 2.5 mmol/L) on Pb(II) (C_0_ = 15 mg/L) adsorption by PAFB. The molar concentration ratio of coexisting ions to Pb(II) is 104.17: 1. All coexisting-ion experiments are conducted in a background electrolyte of 0.01 mol/L NaNO_3_, and the pH is adjusted with HNO_3_/NaOH. All coexisting-ion experiments are performed in triplicate. The relative standard deviation of adsorption data (0.07% ≤ RSD ≤ 4.81%) indicates that the results are statistically significant.

Without the addition of coexisting ions, the equilibrium adsorption capacity (*Q*_e_) of PAFB for Pb(II) is 13.38 mg/g. After the introduction of coexisting ions, the *Q*_e_ values for Pb(II) varied to different degrees in each system. Specifically, the *Q*_e_ values in the Ca^2+^ and Mg^2+^ groups are 12.81 mg/g and 14.56 mg/g, respectively, which are roughly comparable to the blank value of 13.2 mg/g. This indicates that although typical alkali-metal cations (Ca^2+^ and Mg^2+^) exhibit certain electrostatic competition with Pb(II), their interference effect is not significant [8,17]. This can be attributed to the higher coordination selectivity of abundant oxygen-containing functional groups and Mn–OH sites on the PAFB surface toward Pb(II). In the system with coexisting Mn^2+^, the *Q*_e_ value decreases to 6.41 mg/g with a reduction of 52.09%. This may be attributed to the similar ionic radius and coordinated chemical behavior between Mn^2+^ and Pb^2+^, so that strong competitive adsorption is formed between them at active sites such as ≡Mn–OH and –COO^−^ [48,60]. In the system containing coexisting ${SO}_{4}^{2-}$ the *Q*_e_ value decreases to 3.53 mg/g with a reduction of 73.62%. This may be related to the divalent property of ${SO}_{4}^{2-}$. It can interact more strongly with divalent Pb(II) in solution to form water-soluble Pb–SO_4_ complexes, reducing the concentration of free Pb^2+^ available for adsorption [61,62]. Meanwhile, sulfate ions may compete for the binding sites of Pb(II), especially those associated with Mn–OH/Mn–O groups [62]. In the system with coexisting ${HPO}_{4}^{2-}$, the *Q*_e_ value drops sharply to 1.05 mg/g with a high reduction of 92.15%, exhibiting the strongest inhibitory effect among all tested coexisting ions. The core reason for this phenomenon is that a strong chemical precipitation reaction occurred between ${HPO}_{4}^{2-}$ and Pb^2+^, fundamentally altering the occurrence species of Pb(II) in the solution [63].

Accordingly, the inhibition sequence of coexisting ions toward Pb(II) adsorption by PAFB is ranked as ${HPO}_{4}^{2-}$ > ${SO}_{4}^{2-}$ > Mn^2+^ >Ca^2+^ ≈ Mg^2+^, indicating that the adsorption of Pb(II) by PAFB is more sensitive to strongly coordinating anions or divalent anions.

#### 2.2.4. Effects of Contact Time

Figure 9 shows the effects of contact time (5–2880 min) on the time adsorption capacity (*Q*_t_) and removal efficiency (*R*_t_) of PAFB for Pb(II). The adsorption process of Pb(II) onto PAFB exhibits a distinct two-stage characteristic. In the initial fast-adsorption stage (0–60 min), both *Q*_t_ and *R*_t_ values increase approximately linearly with time. At 60 min, the *Q*_t_ value reaches 8.88 mg/g with an *R*_t_ value of 85.69%. This can be attributed to the abundant vacant active sites available on the PAFB surface at the early adsorption stage. A large concentration gradient of Pb(II) in the solution provides a strong driving force, and Pb(II) is rapidly captured by surface active sites. In the subsequent slow-growth stage (60–2280 min), the *Q*_t_ value increases slowly from 8.88 mg/g to 9.67 mg/g, and the *R*_t_ value rises from 85.69% to 93.38%, while the adsorption rate is obviously reduced. This is because the surface-active sites are gradually saturated. The remaining unoccupied sites are mostly located inside pores or belong to low-energy adsorption sites, and greater diffusion resistance needs to be overcome for Pb(II), so the adsorption gradually approaches dynamic equilibrium [23]. Notably, the *Q*_t_ value reaches 9.58 mg/g (*R*_t_ value of 92.50%) at 1440 min (24 h). Only a limited increment of 0.09 mg/g is obtained from 1440 min to 2880 min (48 h). This indicates that the adsorption system basically reaches equilibrium after 24 h. Accordingly, 24 h (1440 min) is selected as the optimal equilibrium time for the subsequent isotherm and thermodynamic experiments.

#### 2.2.5. Effect of Initial Concentration and Temperature

Figure 10 shows the effects of initial Pb(II) concentration (*C*_0_, 5–300 mg/L) and temperature (25 °C, 35 °C, and 45 °C) on the equilibrium adsorption capacity (*Q*_e_) of PAFB for Pb(II). The *Q*_e_ value increases rapidly at first and then levels off with the rise of *C*_0_ under all three temperature conditions. In the low-concentration range (0–150 mg/L), *Q*_e_ values increase almost linearly with increasing *C*_0_. This can be explained by the fact that the active sites on PAFB are not sufficiently saturated. A higher concentration gradient in aqueous solution provides a stronger mass-transfer driving force, which promotes the migration and immobilization of Pb(II) onto the adsorbent [23,64]. When *C*_0_ exceeds 150 mg/L, the growth rate of *Q*_e_ is obviously reduced and gradually approaches equilibrium, indicating that the active sites on PAFB are steadily saturated, and the adsorption approaches the maximum capacity. Temperature exhibits an obvious positive promoting effect on adsorption performance. At *C*_0_ = 250 mg/L, the *Q*_e_ values at 25 °C, 35 °C, and 45 °C are 50.84 mg/g, 56.05 mg/g, and 61.05 mg/g, respectively. The adsorption capacity improves with increasing temperature, demonstrating that the adsorption of Pb(II) by PAFB is an endothermic process, and higher temperature is beneficial to the improvement in adsorption capacity.

### 2.3. Adsorption Characteristics

#### 2.3.1. Adsorption Kinetics

Figure 11 shows the linear fitting curves of pseudo-first-order (a) and pseudo-second-order (b) kinetic models achieved via Equations (3) and (4), respectively, and Table 1 summarizes the corresponding kinetic parameters. The fitting curve of the pseudo-first-order kinetic model exhibits a poor linear relationship, with a determination coefficient (*R*^2^) of only 0.2953. Moreover, the theoretical equilibrium adsorption capacity (*Q*_e, cal_, 7.08 mg/g) calculated by this model deviates greatly from the experimental value (*Q*_e, exp_, 9.67 mg/g), indicating that the pseudo-first-order kinetic model is not suitable for describing the adsorption process of Pb(II) onto PAFB. In contrast, the fitting curve of the pseudo-second-order kinetic model presents an excellent linear relationship (*R*^2^ = 0.9997). The theoretical (*Q*_e, cal_, 9.74 mg/g) value calculated from this model is much closer to the experimental value, demonstrating that the adsorption rate of Pb(II) by PAFB is limited by chemical interactions such as electron sharing or exchange between active sites on adsorbent surfaces and adsorbates rather than by the physical diffusion process [65,66].

#### 2.3.2. Adsorption Isotherms

Figure 12 shows the fitting curves of the Langmuir (a) and Freundlich (b) isotherm models for Pb(II) adsorption by PAFB, and Table 2 summarizes the corresponding isotherm parameters. At 25 °C, 35 °C, and 45 °C, the equilibrium adsorption capacity (*Q*_e_) of PAFB for Pb(II) increases with the rise in equilibrium concentration (*C*_e_). With the same *C*_e_ value, *Q*_e_ values increase as temperature rises, which further demonstrates that the adsorption process is endothermic [67]. In terms of determination coefficients, the *R*^2^ values of the Freundlich model at three temperatures are 0.8789 (25 °C), 0.9101 (35 °C), and 0.9363 (45 °C), respectively, which are higher than those of the Langmuir model (0.8318, 0.8250, and 0.8307). This indicates that the Freundlich model can better describe the adsorption behavior of Pb(II) onto PAFB. This result reveals that active sites with heterogeneous energy distribution exist on the PAFB surface [37,68]. The adsorption of Pb(II) on PAFB is not an ideal homogeneous monolayer adsorption, but involves multi-layer coverage or synergistic effects among sites with different affinities. The heterogeneity factor *n* of the Freundlich model is 4.7654, 4.4006, and 4.2517 at 25 °C, 35 °C, and 45 °C, respectively. All *n* values are much greater than 1, indicating that the adsorption process of Pb(II) onto PAFB proceeded spontaneously. Moreover, the *n* value decreases slightly with increasing temperature, implying that the energy distribution of adsorption sites becomes more diverse under high-temperature conditions [37,69]. The theoretical maximum *Q*_e_ fitted by the Langmuir model increases from 45.62 mg/g (25 °C) to 49.62 mg/g (35 °C) and 53.87 mg/g (45 °C) with the increase in temperature, which further confirms the endothermic adsorption characteristic. Accordingly, the adsorption of Pb(II) by PAFB follows a composite adsorption mechanism dominated by chemical complexation and characterized by multi-layer adsorption on heterogeneous surfaces. This is highly consistent with the diversity of surface oxygen-containing functional groups (–OH, –COOH) and ≡Mn–OH sites revealed by the aforementioned XPS and FTIR analyses.

#### 2.3.3. Thermodynamic Characteristics

Figure 13 shows the Van’t Hoff linear fitting curve for Pb(II) adsorption onto PAFB, and the corresponding thermodynamic parameters are summarized in Table 3. A satisfactory linear relationship (*R*^2^ = 0.9535) is obtained in the temperature range of 298.15 K–318.15 K, demonstrating that the adsorption behavior of Pb(II) onto PAFB conforms to the basic assumptions of the Van’t Hoff equation within this temperature interval. As shown in Table 3, the positive standard enthalpy change (Δ*H*^θ^ = 26.33 kJ/mol) confirms that the adsorption of Pb(II) onto PAFB is an endothermic process, and higher temperatures facilitate the adsorption reaction [68]. The positive value of Δ*S*^θ^ (112.57 J/(mol·K)) suggests an increase in disorder at the solid–liquid interface during adsorption. Meanwhile, this positive value reflects the favorable affinity between the adsorption sites on the PAFB surface and Pb(II) [68]. The values of Δ*G*^θ^ are negative at all three temperatures (−7.30 kJ/mol, −8.20 kJ/mol, and −9.56 kJ/mol at 298.15 K, 308.15 K, and 318.15 K, respectively) and decrease monotonously with increasing temperature. These results verify that the adsorption of Pb(II) onto PAFB is a spontaneous process, and higher temperature can effectively improve the spontaneity and driving force of adsorption.

### 2.4. Regeneration of PAFB Desorption of Pb(II)

Figure 14 shows the effect of the adsorption–desorption cycle number on Pb(II) removal efficiency (*R*_e_) by PAFB. The PAFB achieves an *R*_e_ value of 93.15% in the first adsorption. After desorption and regeneration with 50 mL of 0.5 mol/L ammonia solution, the *R*_e_ value decreases to 90.29% for the first regeneration cycle and to 87.13% for the second cycle, remaining at approximately 87.28% for the third and fourth cycles. After four successive adsorption–desorption cycles, the *R*_e_ value of PAFB only drops from 93.15% to 87.26%, with a total reduction of merely 5.89%. These results demonstrate that PAFB possesses favorable regeneration performance and reusability stability. The slight decline in adsorption efficiency may be attributed to the variation in surface functional groups, which weakens the complexation affinity toward Pb(II) [23]. Notably, the removal efficiency stays stable at around 87.0% from the second to the fourth cycle. This indicates that a considerable number of effective adsorption sites are still retained on PAFB even after multiple regeneration treatments, and stable adsorption performance can be maintained. Overall, PAFB exhibits excellent reusability, which provides critical support for its economical application in the practical treatment of Pb(II)-containing wastewater.

### 2.5. Performance Evaluation

Table 4 shows the adsorption performance of PAFB for Pb(II) removal using various other biochar-based materials. The Langmuir fitting results show that the maximum adsorption capacity (*Q*_m_) of PAFB for Pb(II) is 45.62 mg/g at 25 °C and pH = 6.0, which is higher than that of magnetic γ-Fe_2_O_3_-loaded sludge biochar (35.27 mg/g) [14], peanut shell biochar (42.74 mg/g) [12], pine wood biochar (20.30 mg/g) [18], corn stalk biochar (31.30 mg/g) [15], and coconut shell biochar (32.31 mg/g) [23], demonstrating the favorable adsorption performance of PAFB. Nevertheless, the adsorption capacity of PAFB is lower than that of Zn-modified coconut shell biochar (84.25 mg/g) [23], coffee ground biochar (428.60 mg/g) [16], and jackfruit peel biochar (83.86 mg/g) [22], indicating that there is still considerable room for further improvement in the adsorption capacity of PAFB.

The advantages of PAFB lie in the wide availability and low cost of areca fiber raw materials, as well as the simple and scalable KMnO_4_ modification process. Moreover, PAFB maintains a Pb(II) removal efficiency of approximately 87.00% after four adsorption–desorption cycles, exhibiting excellent reusability and operational stability. The main limitations of PAFB are its relatively low specific surface area (11.06 m^2^/g), which restricts the contribution of physical adsorption, and its moderate maximum adsorption capacity (45.62 mg/g) compared with that of most biochar adsorbents reported in previous studies.

Accordingly, future optimization directions are proposed as follows: (1) The pyrolysis and activation conditions (e.g., elevated pyrolysis temperature, CO_2_ or steam activation) can be optimized to improve the specific surface area and pore volume of PAFB. (2) Composite modification strategies combining KMnO_4_ with other modifiers (e.g., FeCl_3_ and ZnCl_2_) can be explored to increase the density of surface active sites. (3) The loading amount of MnO_2_ can be systematically optimized to achieve a balance between the enhancement of active sites and the avoidance of pore blockage. (4) PAFB can be further applied to dynamic column adsorption experiments using real industrial wastewater to evaluate its feasibility for practical engineering applications.

### 2.6. Adsorption Mechanism

Combining the characterization results of XRD, FTIR, and XPS with adsorption experimental data, the mechanism of adsorption of Pb(II) onto PAFB is systematically summarized as the dual mechanisms of electrostatic attraction and surface complexation (Figure 15). The electrostatic attraction serves as the initial driving force for the adsorption process. Zeta-potential analyses reveal that PAFB is negatively charged over the pH range of 3.0–7.0 (−14.97 mV to −27.23 mV), which generates electrostatic attraction toward positively charged Pb(II). Moreover, the negative surface charge is intensified with increasing pH, which is highly consistent with the trend that the removal efficiency increases significantly with rising pH (85.74% at pH = 3.0; 96.16% at pH = 6.0).

On the other hand, surface complexation acts as the core pathway for adsorption. XRD results verify that MnO_2_ nanoparticles are successfully loaded onto the biochar surface after KMnO_4_ modification, providing abundant inorganic ≡Mn–OH active sites for Pb(II) adsorption (Figure 3). The FTIR and XPS C1s/O1s spectra further confirm that PAFB is rich in oxygen-containing functional groups, including –OH, –COOH, and C=O (Figure 4). After adsorption, the intensities of characteristic peaks for –OH (3425 cm^−1^) and C=O (1631 cm^−1^) in FTIR spectra decrease remarkably. In the XPS C1s spectrum, the C–O peak shifts toward lower binding energy, and its relative content drops to 24.52%. Meanwhile, the content of C=O decreases from 13.14% to 10.38% (Figure 5a). The content of M–OH in O1s increases from 29.03% to 30.43%, demonstrating that Pb^2+^ forms inner-sphere complexes (≡C–O–Pb^+^, ≡C(=O)–O–Pb^+^, ≡Mn–O–Pb^+^) via ligand exchange with oxygen atoms from –OH, –COOH, and ≡Mn–OH sites (Figure 5c). Only characteristic peaks corresponding to complexed Pb(II) are detected at 138.15 eV and 143.24 eV in the XPS Pb4f spectrum, while no Pb^0^ signal is observed. This further confirms that Pb(II) is stably immobilized in the form of amorphous surface complexes rather than through reduction or precipitation pathways (Figure 5e).

The synergistic effect of electrostatic attraction and surface complexation endows PAFB with favorable adsorption performance toward Pb(II). Even though its specific surface area is only 11.06 m^2^/g, efficient Pb(II) removal can still be achieved owing to the presence of abundant chemically active sites (–OH, –COOH, and ≡Mn–OH).

## 3. Experimental Methods

### 3.1. Materials

Raw areca shell materials were purchased from Jiangxi Jingchen Biotechnology Co., Ltd. (Jiangxi, China). Analytical reagent (AR) potassium permanganate (KMnO_4_) was purchased from Chengdu Best Reagents Co., Ltd. (Chengdu, China). Lead nitrate (Pb(NO_3_)_2_, AR) is the source of Pb(II) in aqueous solution and was purchased from Xilong Scientific Co., Ltd. (Shantou, China). Other AR-grade reagents (including HCl and NaOH, etc.) were purchased from Xilong Science Co., Ltd. (Shantou, China). Ultrapure water used in the experiments was prepared using the Milli-Q water purification system (Millipore Corporation, Burlington, MA, USA).

### 3.2. Preparation of PAFB Adsorption Material

(1) Pretreatment of areca shell

A total of 500 g of raw areca shells were thoroughly rinsed with ultrapure water to remove surface impurities and subsequently soaked in 20% NaOH solution for 72 h. After repeated cleaning, the samples were dried in an oven at 50 °C to a constant weight and then removed. The outer shells were peeled off to obtain pure areca fibers.

(2) Preparation of PAFB material

A total of 30 g of areca fiber was weighed and placed in a crucible, which was then loaded into an unheated tube furnace. Pyrolysis was carried out at 300 °C for 5 min under an N_2_ flow rate of 50 mL/min and a heating rate of 5 °C/min. After the muffle furnace cooled to room temperature, bulk AFB was obtained. Note: when the pyrolysis time exceeded 5 min, the areca fiber was fully carbonized and unfavorable for sample recovery. Subsequently, AFB was completely immersed in 50 mL of 0.2 mol/L KMnO_4_ solution, sealed, and placed in a water-bath shaker operated at 25 °C and 170 r/min for 24 h. After the immersion was completed, the treated AFB was removed and dried to constant weight in an oven at 50 °C, and the KMnO_4_-modified areca fiber biochar was finally obtained.

### 3.3. Batch Adsorption Experiment

The batch experiments were aimed at investigating the effects of modification treatment and environmental factors on the adsorption performance of the materials. The former mainly explored the modification performance of potassium permanganate on areca fiber biochar under different pH conditions (2.0–7.0). The latter investigated the effects of various environmental factors, including pH conditions (2.0–7.0), solid–liquid ratio (*m*/*v*, 0.5–3.0 g/L), coexisting ions (${HPO}_{4}^{2-}$, ${SO}_{4}^{2-}$, ${NO}_{3}^{-}$, Ca^2+^, Mn^2+^, and Mg^2+^, *C*_0_ = 2.5 mmol/L), contact time (*t*, 5–2880 min), the Pb(II) initial concentration (*C*_0_, 5–300 mg/L), and temperature (25 °C, 35 °C, and 45 °C), on the adsorption performance of PAFB. Taking the pH effect experiment as an example, 50 mg of AFB and/or PAFB was added to a series of 100 mL polyethylene centrifuge tubes, followed by the addition of 50 mL of 15 mg/L Pb(II) solution.

Subsequently, the initial pH of each batch solution was adjusted to the desired value (2.0–7.0) using 0.1 mol/L HNO_3_ and NaOH. The centrifuge tubes were sealed and placed in a constant-temperature water bath oscillator and stirred at a speed of 180 r/min and 25 ± 1 °C for 48 h (preliminary experiments have confirmed that adsorption equilibrium is reached within this period). After stirring, the supernatant was collected to determine the concentration of Pb(II). All the aqueous samples before and after adsorption were filtered through a 0.45 μm water-based membrane filter, and the concentration of Pb(II) in the water phase was determined by inductively coupled plasma optical emission spectrometry (Optima 7000 DV, Platinum Elmer Instruments, Inc., Waltham, MA, USA).

The equilibrium adsorption capacity (*Q*_e_) and removal efficiency (*R*_e_) of PAFB and AFB for Pb(II) are calculated using the formulas below:

(1) $$Q_{e}=\frac{\left(C_{0}-C_{e}\right)\cdot V}{m}$$

(2) $$R_{e}=\frac{\left(C_{0}-C_{e}\right)}{C_{0}}\times 100\%$$

where *Q*_e_ is the adsorption capacity of PAFB and AFB for Pb(II) at adsorption equilibrium, mg/g. *C*_0_ is the initial Pb(II) concentration, mg/L. *C*_e_ is the concentration of Pb(II) at adsorption equilibrium, mg/L. *V* is the volume of simulated liquid containing Pb(II), L. *m* is the dosage of adsorbent, g. *R*_e_ is the removal efficiency of Pb(II) at adsorption equilibrium, %.

Subsequently, adsorption kinetics, isotherms, and thermodynamics were investigated under the optimal pH and solid-to-liquid ratio to further evaluate the adsorption performance of the PAFB material.

Three parallel groups were established in each experiment. The experimental results and graphs are expressed as the mean and relative standard deviation (RSD, %), and the RSD is less than 5%.

The experimental results were expressed as the average of three groups of data, and the relative standard deviation (RSD, %) between the data was less than 5%. Meanwhile, the data chart is drawn with error bars showing the average and RSD.

### 3.4. Adsorbent Regeneration

It has been reported that the adsorbent is regenerated and desorbed with 0.1 mol/L or 10 wt.% ammonia solution [45,70]. Ammonia solution is a weak alkali and imposes lower corrosiveness on the activated carbon skeleton and metal-modified parts. Moreover, ammonium ions can compete with heavy metals (such as Cd, Cr, Pb, and Zn, etc.) for cation exchange sites on the adsorbents [71]. Meanwhile, when the concentration of ammonium ions is high, this competitive effect is especially significant. Therefore, in this study, after the adsorption experiment, the PAFB adsorbed with Pb(II) was desorbed and regenerated using 50 mL of 0.5 mol/L ammonia. The centrifuge tube was placed into a constant-temperature water bath oscillator at 25 °C and 180 rpm for 1440 min. Then, the Pb(II) concentration in the desorption solution was determined by ICP-OES. Subsequently, the regenerated PAFB was thoroughly rinsed with ultra-pure water until the washing effluent was nearly neutral and then dried at 45 °C in a muffle furnace for later use. This adsorption–desorption cycle was repeated four times. After adsorption and regeneration, the bulk adsorbent was separated from the solution by filtration [45,70].

### 3.5. Kinetic, Isotherm, and Thermodynamic Models

#### 3.5.1. Kinetic Models

Adsorption kinetics aims to elucidate the adsorption rate and its underlying control mechanisms, thereby distinguishing the respective contributions of physical diffusion and chemical bonding to the overall adsorption process [72]. In this study, the experimental data are fitted using the pseudo-first-order (PFO) and pseudo-second-order (PSO) kinetic models. Among these, the pseudo-first-order (PFO) kinetic model assumes that the adsorption rate is limited by the diffusion of adsorbate molecules to the surface of the adsorbent, and this rate is proportional to the number of unoccupied active sites [65]. Therefore, the model is suitable for describing the physical adsorption or rapid adsorption process in the initial stage. The equation is as follows:

(3) $$\log(Q_{e}-Q_{t})=\log Q_{e}-\frac{K_{1}}{2.303}t$$

The pseudo-second-order (PSO) kinetic model assumes that the adsorption rate is controlled by chemical interaction (such as electron sharing or exchange) between the adsorbate and active sites on the adsorbent surface, and the rate is proportional to the square of the active site occupation rate [65,73]. Therefore, this model is usually used to describe the chemisorption process involving chemical bond formation. The equation is as follows:

(4) $$\frac{t}{Q_{t}}=\frac{1}{K_{2}Q_{e}^{2}}+\frac{t}{Q_{e}}$$

where *Q*_e_ and *Q*_t_ are the removal capacities of Pb(II) at adsorption equilibrium and time (*t*), respectively, mg/g. *K*_1_ and *K*_2_ are the adsorption rate constants of pseudo-first-order and pseudo-second-order kinetics, respectively.

#### 3.5.2. Isotherm Models

This study selected two isotherm models (Langmuir and Freundlich) to fit the equilibrium data. The Langmuir isotherm model assumes of an ideal homogeneous surface, wherein the adsorbent possesses a finite number of energetically equivalent active sites, with no lateral interactions among adsorbed molecules, and each site accommodates only one adsorbate molecule [66]. The model allows the theoretical maximum monolayer adsorption capacity to be estimated, and its expression is as follows:

(5) $$\mathrm{Langmuir}:\;Q_{e}=\frac{Q_{m}K_{L}C_{e}}{1+K_{L}C_{e}}$$

The Freundlich isotherm model is an empirical model applicable to multilayer adsorption on heterogeneous surfaces [69]. It reflects the exponential energy distribution of adsorption sites and provides insight into the adsorption intensity. Its expression is given as

(6) $$\mathrm{Freundlich}:\;Q_{e}=K_{F}C_{e}^{1/n}$$

where *Q*_e_ is the adsorption capacity of PAFB for Pb(II) at adsorption equilibrium, mg/g. *C*_e_ is the mass concentration of Pb(II) in the solution at adsorption equilibrium, mg/L. *Q*_m_ is the maximum adsorption capacity for Pb(II), mg/g. *K*_L_ is the Langmuir adsorption equilibrium constant, which is related to the strength of the adsorption interaction. *K*_F_ and 1/*n* are the adsorption equilibrium constant and adsorption strength constant of the Freundlich equation, respectively.

#### 3.5.3. Thermodynamic Model

Thermodynamic model can examine the spontaneity, thermal effect, and changes in the degree of disorder at the solid–liquid interface during the adsorption process [68]. The thermodynamic state functions (including the Gibbs free energy change (Δ*G*^θ^), enthalpy change (Δ*H*^θ^), and entropy change (Δ*S*^θ^)) are calculated from temperature-dependent adsorption experiments conducted at 25 °C, 35 °C, and 45 °C. The equations are as follows:

(7) $$∆G^{\theta }=∆H^{\theta }-T∆S^{\theta }$$

(8) $$K_{C}=\frac{C_{0}-C_{e}}{c_{e}}$$

(9) $$\ln K_{c}=-\left(\frac{∆H^{\theta }}{RT}\right)+\frac{∆S^{\theta }}{R}$$

(10) $$∆G^{\theta }=-RTlnK_{c}$$

where Δ*G*^θ^ is the change in Gibbs free energy, kJ/mol. *T* is the temperature measured in Kelvin, K. *R* is the universal gas constant, 8.314 J/(mol·K). *K*_c_ is the adsorbed distribution coefficient. *Q*_e_ is the adsorption capacity at equilibrium, mg/g. *C*_0_ and *C*_e_ are the initial and adsorption equilibrium mass concentrations of Pb(II), respectively, mg/L. Δ*H*^θ^ is the standard enthalpy change, kJ/mol. Δ*S*^θ^ is the standard entropy change, J/(mol K).

### 3.6. Analytical Techniques

PAFB materials obtained before and after adsorption are used for characterization, including BET, XRD, FTIR, SEM–EDS, XPS, etc. In this study, the specific surface area, pore volume, and average pore diameter of AF, AFB, and PAFB are determined by N_2_ adsorption–desorption measurements using an automatic specific surface area and porosity analyzer (BSD-PM4, Beishide Instrument Technology (Beijing) Co., Ltd., Beijing, China). The crystal structure is determined by the X’Pert3 Powder type multifunctional XRD (Panaco, London, UK, Cu target, λ = 1.54056 Å). The scanning step, speed, and range are 0.02626°, 0.6565°/s, and 5–90° (2*θ*), respectively. The functional groups are measured by IS10 FTIR (Thermo Fisher, Waltham, MA, USA). Surface morphology and elemental analysis are determined using JSM–7900F SEM–EDS (JEOL, Tokyo, Japan). The chemical and electronic states of the elements are analyzed by XPS (Escalab 250Xi, Thermo Fisher, Waltham, MA, USA). The zeta potential of PAFB in an aqueous dispersion is measured by the Zetasizer Nano ZS analyzer (Malvern Panalytical, Nano ZS90, Malvern, UK).

## 4. Conclusions

In this study, a biochar composite (PAFB) loaded with MnO_2_ nanoparticles is prepared using areca fiber as the raw material, via potassium permanganate modification. It was found that KMnO_4_ modification greatly improved the adsorption capability of pristine areca-fiber biochar (AFB). The PAFB exhibits superior Pb(II) removal efficiency (*R*_e_) (over 96.00%) compared with that of AFB (less than 1.00%), and thus, PAFB is chosen for subsequent systematic experiments. The adsorption capacity (*Q*_e_) of PAFB for Pb(II) increases from 4.08 mg/g to 13.79 mg/g as pH values rise from 2.0 to 6.0, and the *R*_e_ values increase from 28.45% to 96.16%. With the increase in the solid–liquid ratio (*m*/*v*) from 0.5 g/L to 3.0 g/L, the *Q*_e_ value gradually decreases from 9.12 mg/g to 4.67 mg/g, whereas the *R*_e_ values increase from 31.44% to 96.68%. The *Q*_e_ values grow rapidly and then reach saturation with the elevation of the initial Pb(II) concentration, and the maximum *Q*_e_ value of 61.0 mg/g is obtained at 45 °C. The optimal conditions for Pb(II) are pH = 6.0, *m*/*v* = 1.0 g/L, and an adsorption equilibrium time of 1440 min at 25 °C. The ${SO}_{4}^{2-}$ and ${HPO}_{4}^{2-}$ exert obvious inhibitory effects, while Ca^2+^ and Mg^2+^ show negligible interference. The adsorption of Pb(II) by PAFB conformed to the pseudo-second-order kinetic model (*R*^2^ = 0.9997), where chemisorption plays a dominant role. The Freundlich model provided better fitting results than did the Langmuir model (*R*^2^ = 0.9363 versus *R*^2^ = 0.8318), and *n* > 1 suggests favorable spontaneous adsorption. The thermodynamic parameters (Δ*H*^θ^ = 26.33 kJ/mol and Δ*G*^θ^ < 0) confirm that the adsorption process is spontaneous and endothermic. Characterization analyses of BET, XRD, FTIR, XPS, and zeta potential verified that PAFB possesses a mesopore-dominated porous structure (*S*_BET_ = 11.06 m^2^/g), and MnO_2_ nanoparticles were successfully loaded on biochar surfaces. The Mn–OH sites on MnO_2,_ together with intrinsic oxygen-containing groups (–OH, –COOH, C=O), act as major adsorption sites. PAFB maintained negative zeta potentials (−14.97 mV to −27.23 mV) within pH 3.0–7.0, generating strong electrostatic attraction toward Pb(II). XPS results demonstrate that Pb(II) formed inner-sphere complexes via ligand exchange, and only complexed Pb(II) signals, rather than Pb^0^ or lead-precipitate peaks, are detected. Regeneration experiments reveal that PAFB maintained an approximately 87% removal efficiency after four adsorption–desorption cycles. Ultimately, efficient Pb(II) removal is realized by PAFB through the synergy of electrostatic attraction and surface complexation.

## Figures and Tables

**Figure 1:**
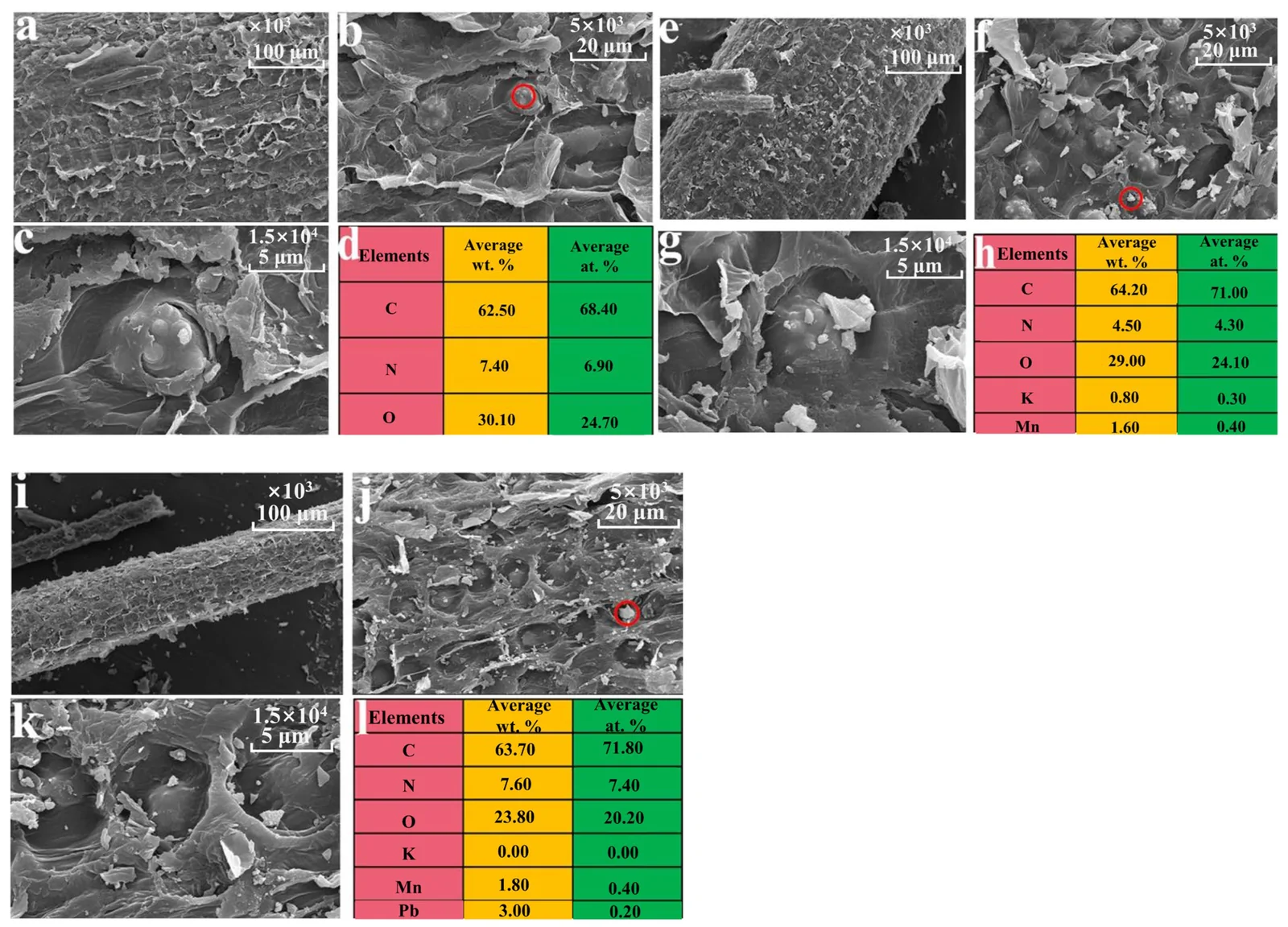
SEM-EDS analysis of AFB (**a**–**d**) and PAFB before (**e**–**h**) and after (**i**–**l**) Pb(II) adsorption.

**Figure 2:**
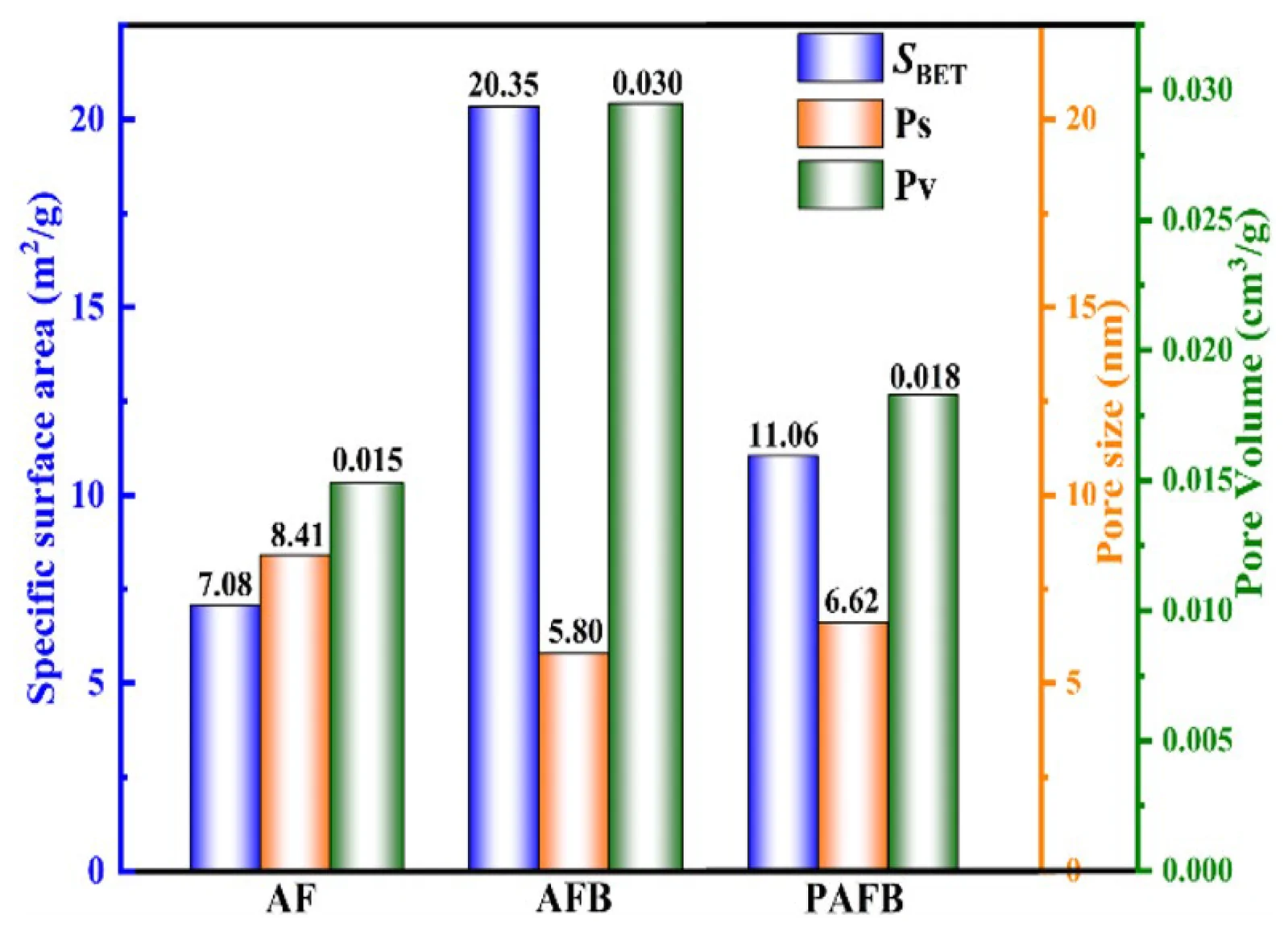
Specific surface area, pore volume, and average pore diameter of AF, AFB, and PAFB.

**Figure 3:**
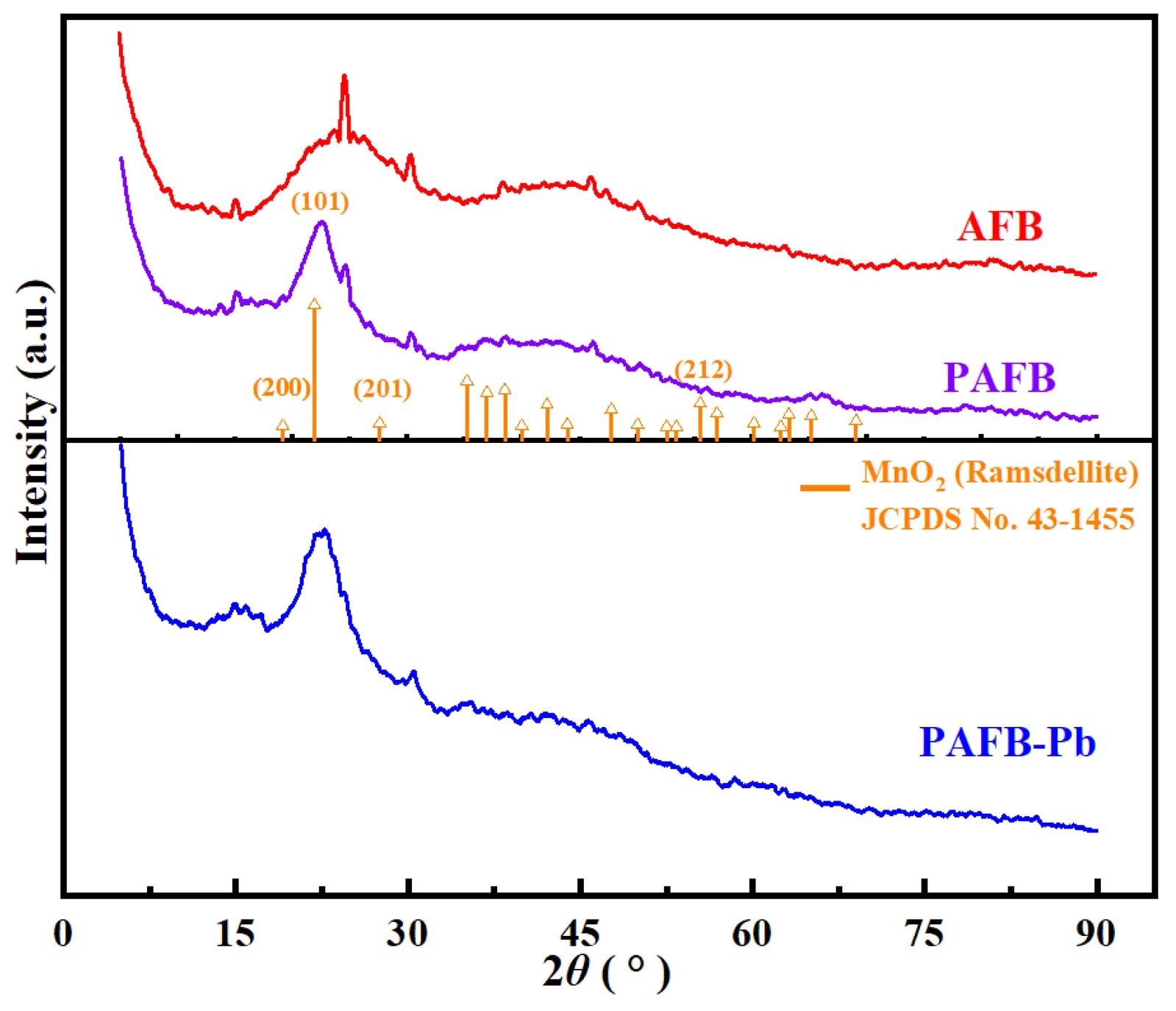
X-ray diffraction (XRD) patterns of AFB, PAFB, and PAFB-Pb.

**Figure 4:**
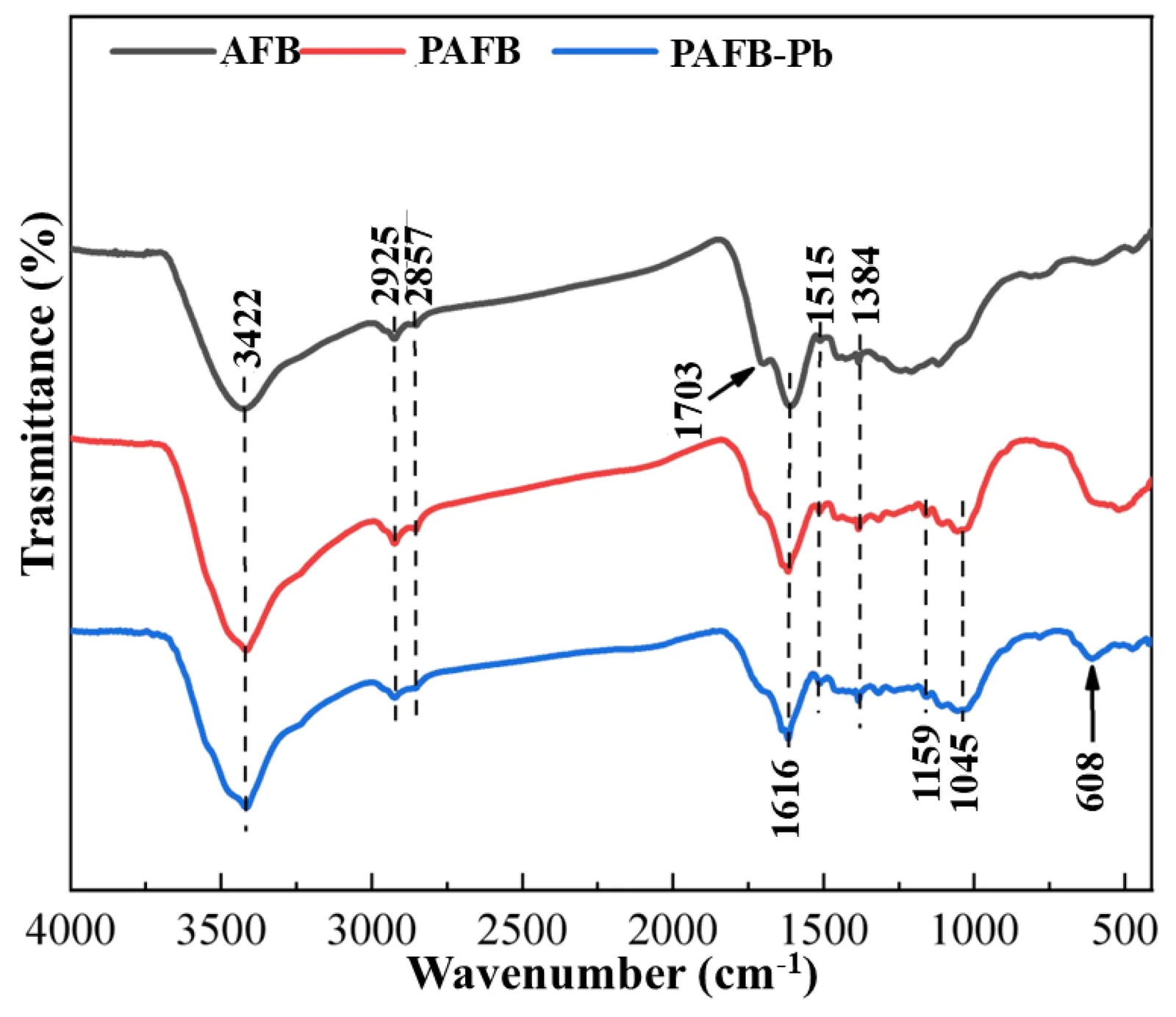
FTIR spectra of AFB, PAFB, and PAFB-Pb.

**Figure 5:**
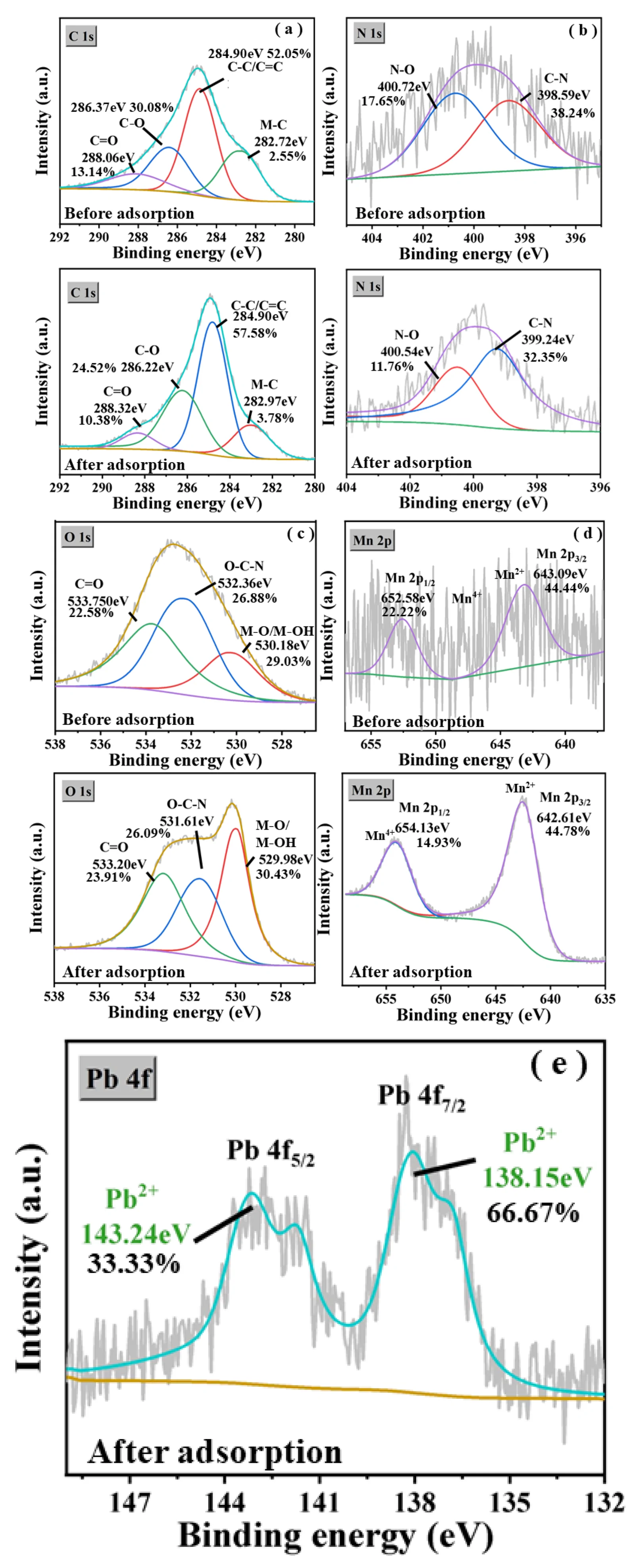
Single-element XPS spectra of C1s (**a**), N1s (**b**), O1s (**c**), Mn2p (**d**), and Pb4f (**e**) for PAFB before and after Pb(II) adsorption.

**Figure 6:**
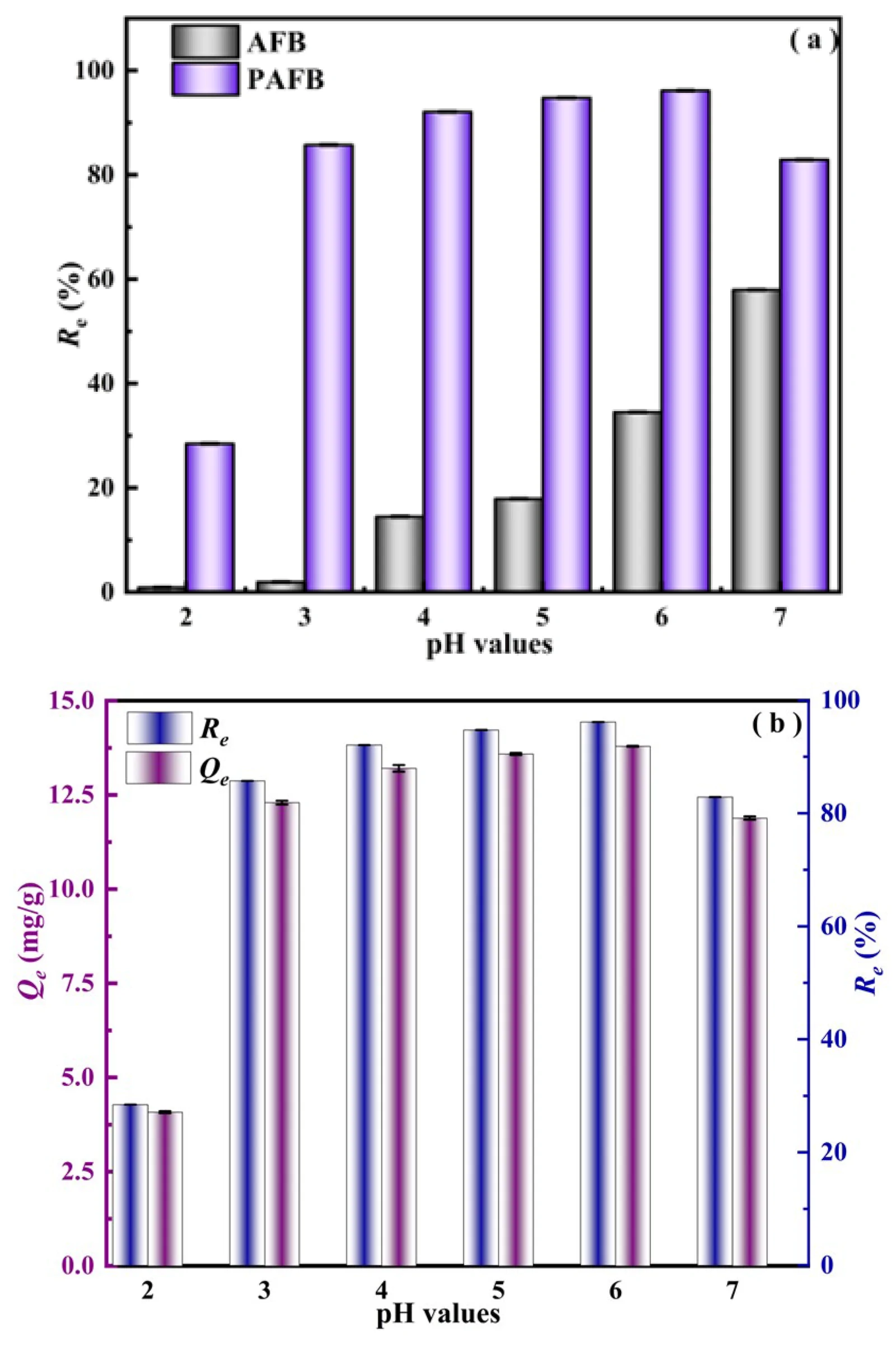
The *R*_e_ values of AFB and PAFB for Pb(II) (**a**) and the *Q*_e_ values of PAFB for Pb(II) (**b**) changed under different pH conditions.

**Figure 7:**
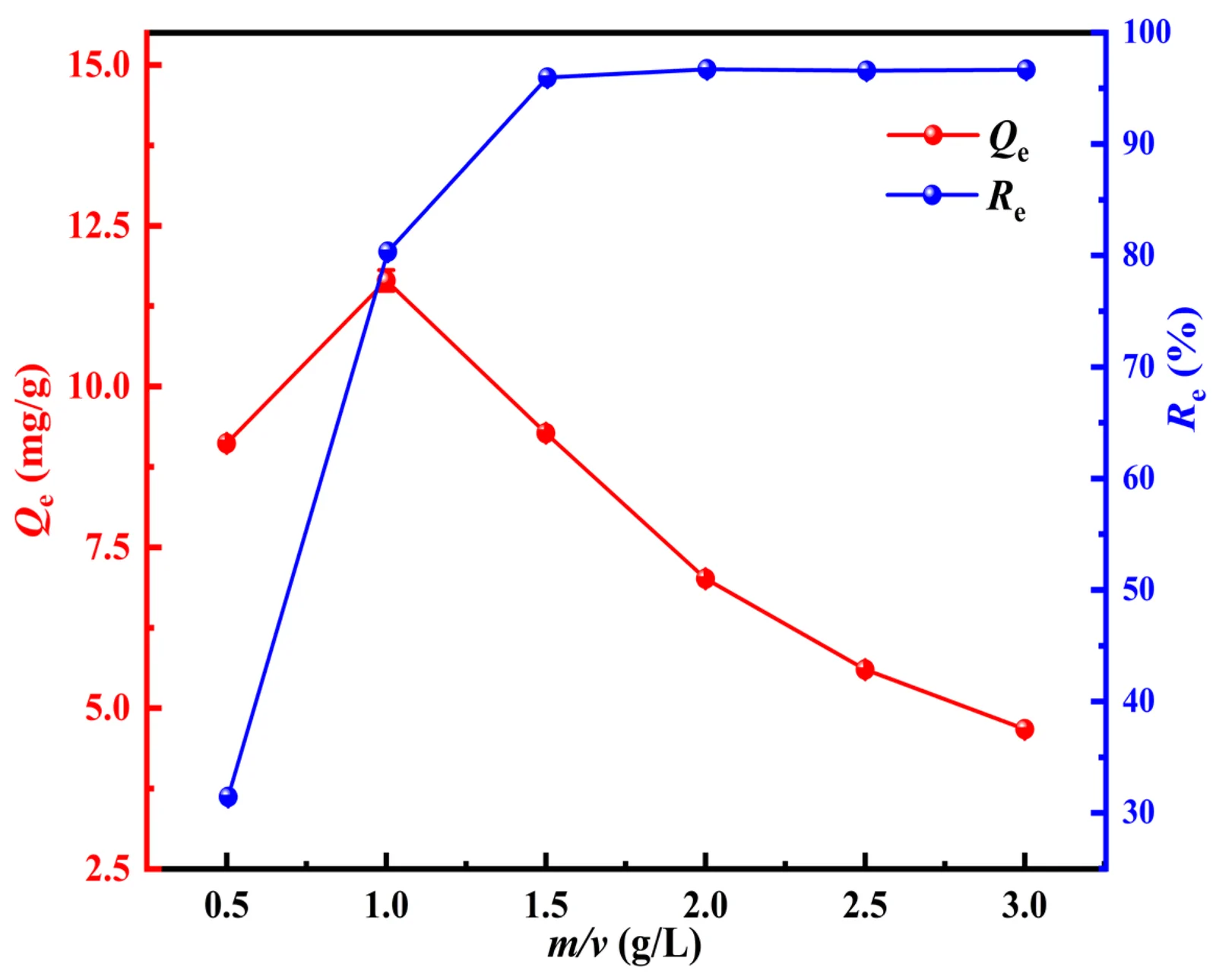
Changes in Pb(II) adsorption capacities (*Q*_e_) and removal efficiency (*R*_e_) with varying *m*/*v* values.

**Figure 8:**
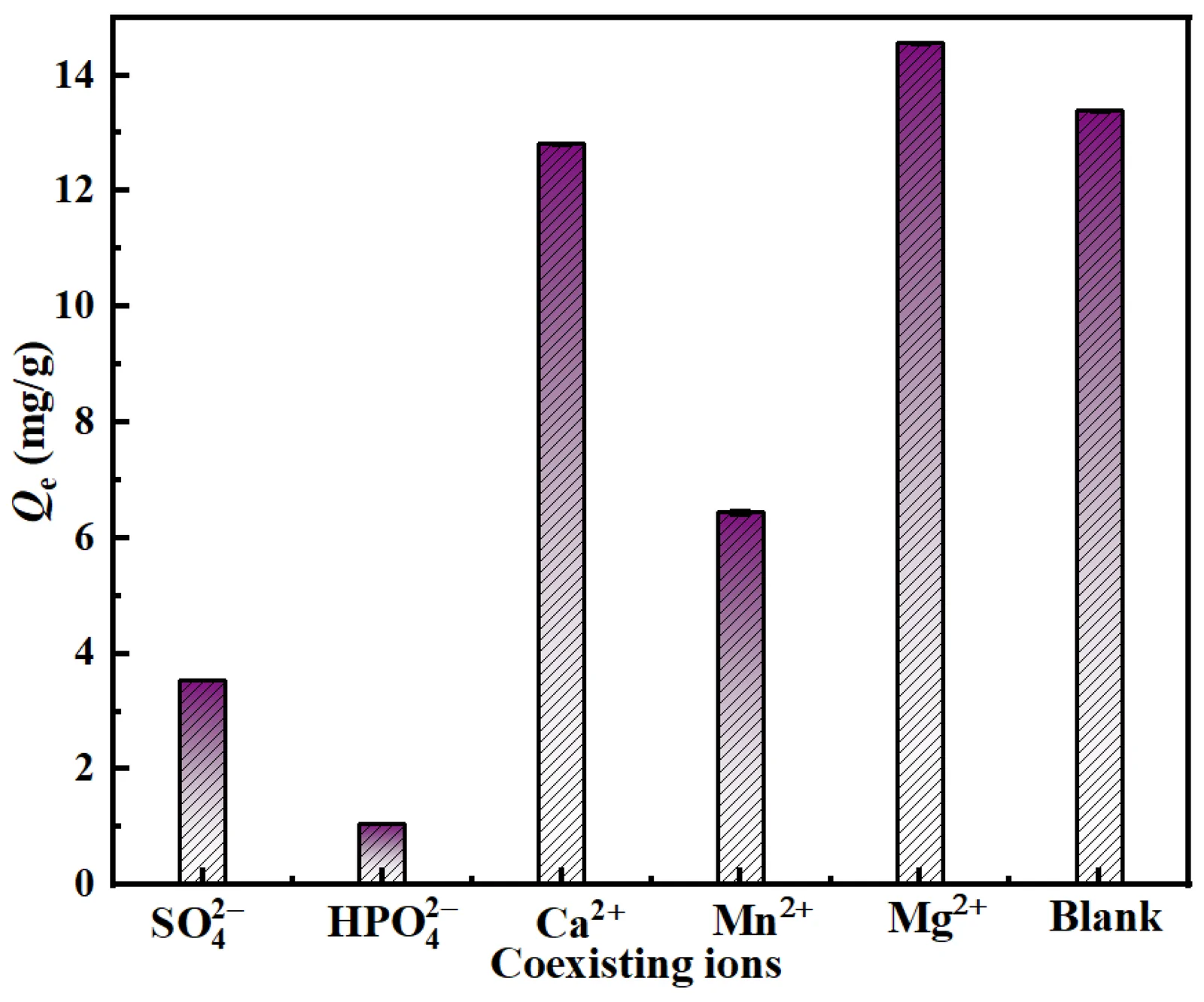
The influence of coexisting ions on the *R*_e_ of Pb(II).

**Figure 9:**
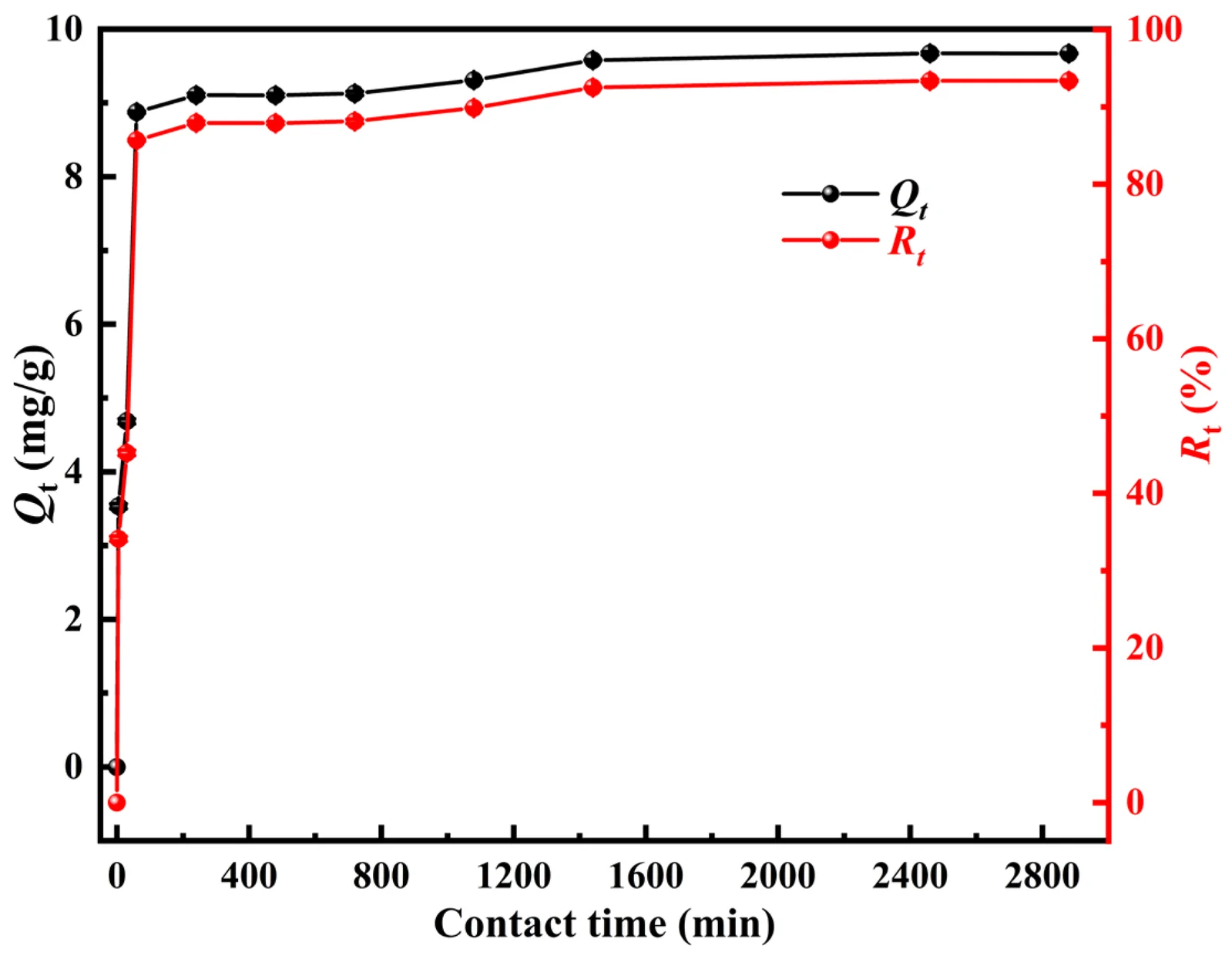
Effect of contact time (*t*) on Pb(II) adsorption by PAFB.

**Figure 10:**
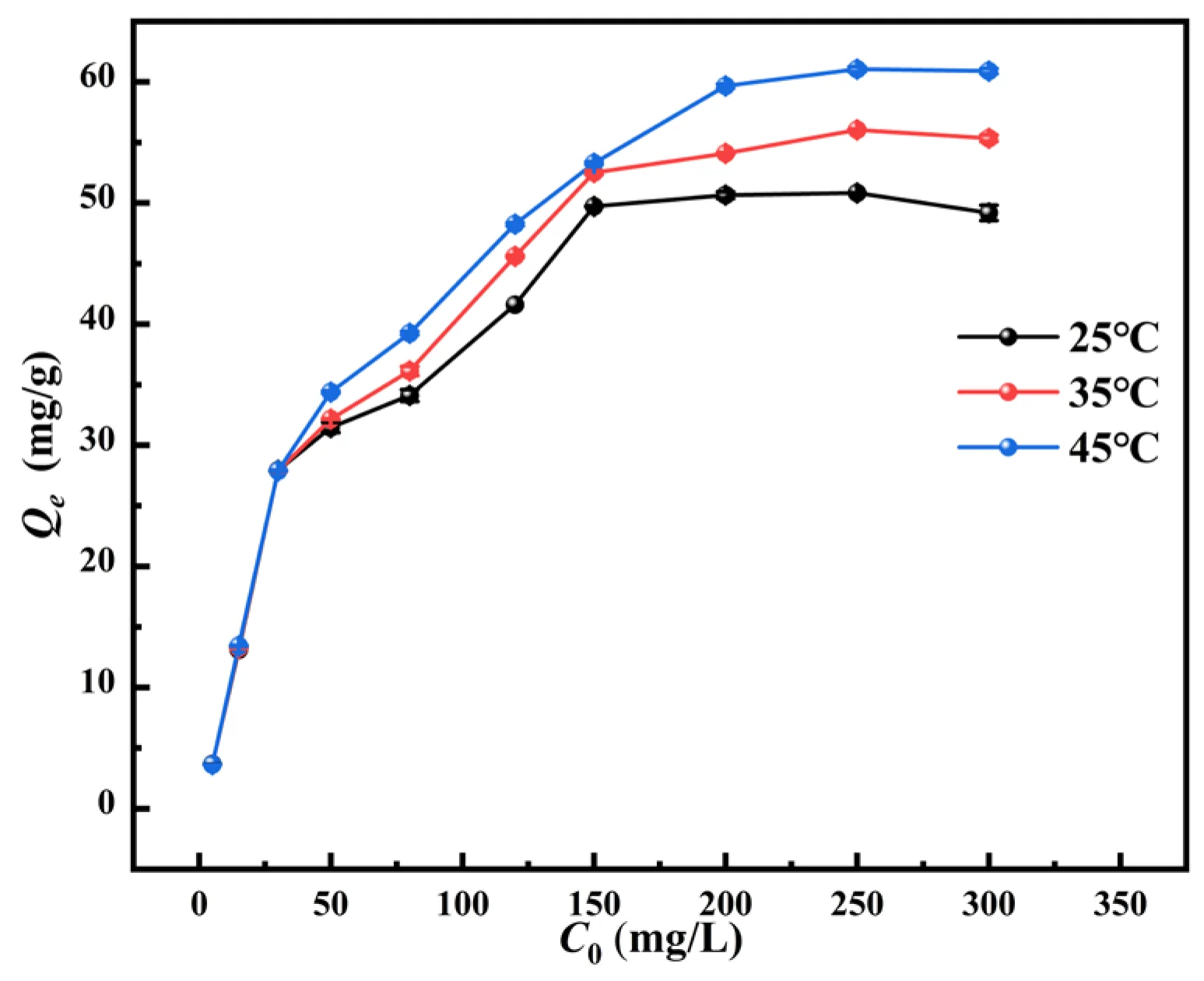
Effect of initial concentration (*C*_0_) and temperature on Pb(II) adsorption by PAFB.

**Figure 11:**
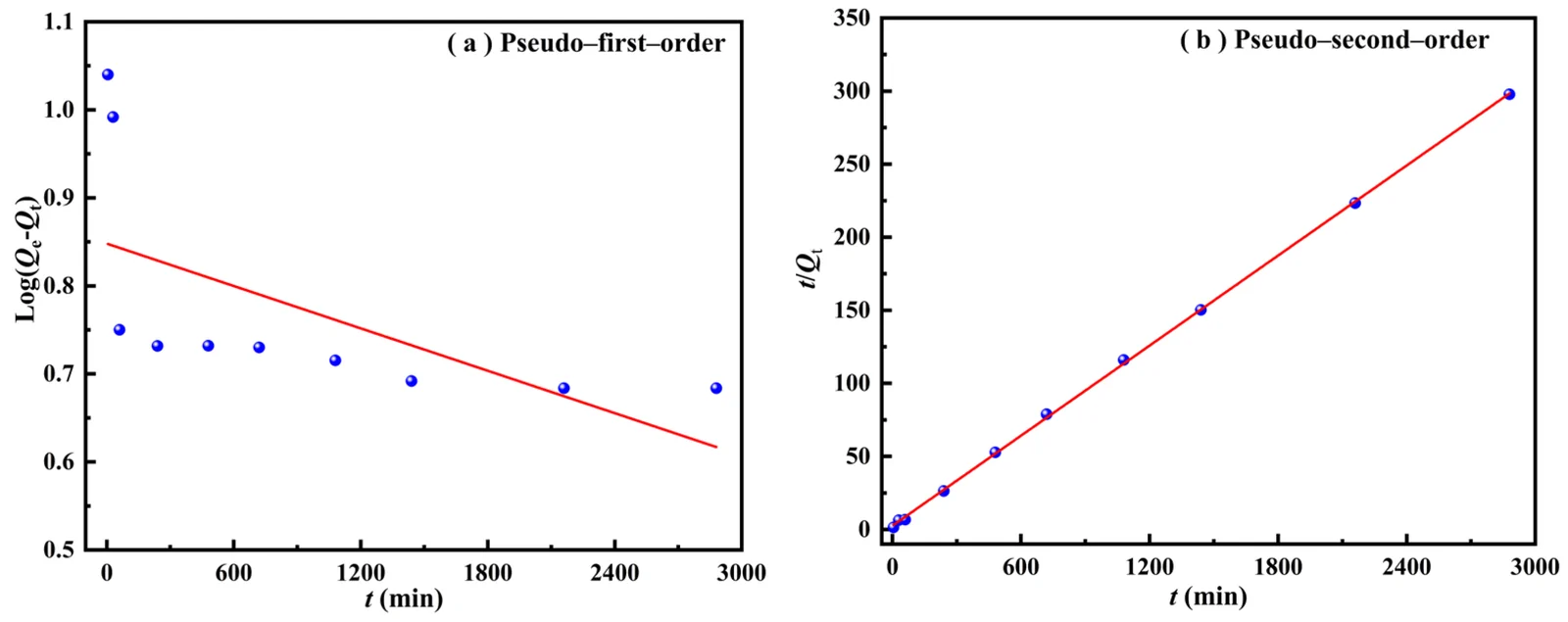
Fitting of the adsorption kinetic model of PAFB adsorption for Pb(II): (**a**) pseudo-first-order kinetic model; (**b**) pseudo-second-order kinetic model.

**Figure 12:**
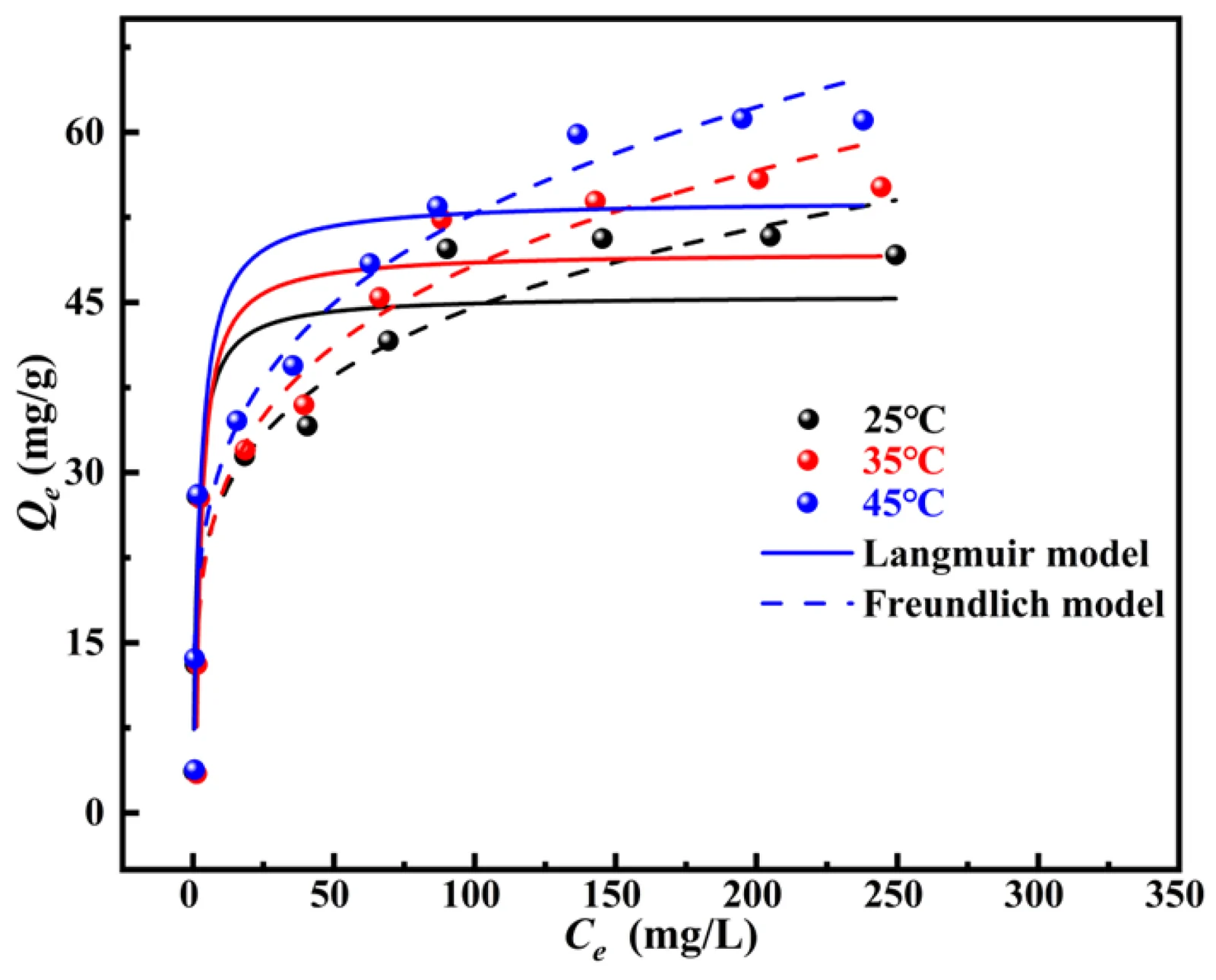
Adsorption isotherm fitting curves of Pb(II) on the composite via the Langmuir and Freundlich models.

**Figure 13:**
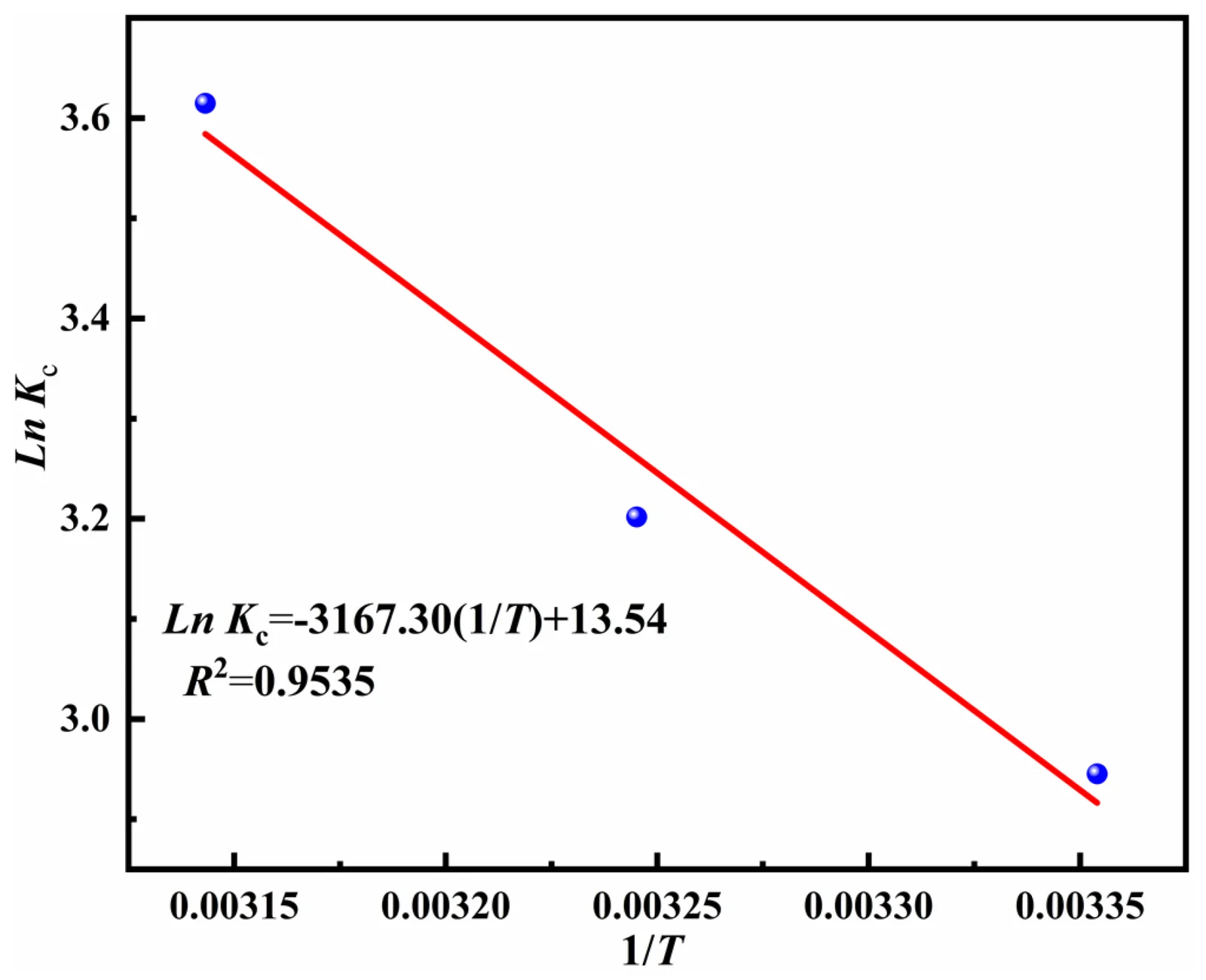
Adsorption thermodynamic curve for Pb(II) adsorption.

**Figure 14:**
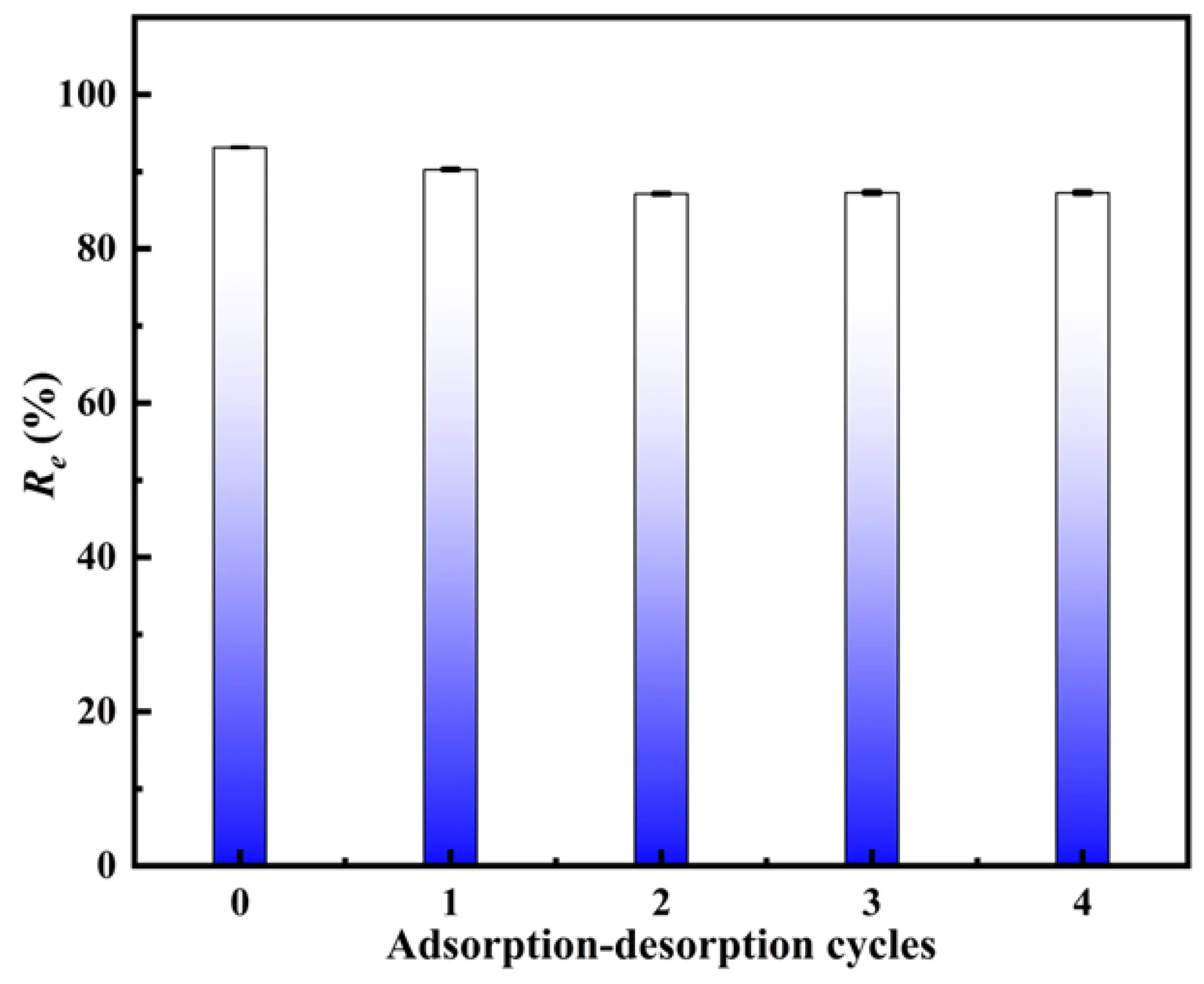
Effect of adsorption–desorption cycle number on the efficiency of Pb(II) removal by PAFB.

**Figure 15:**
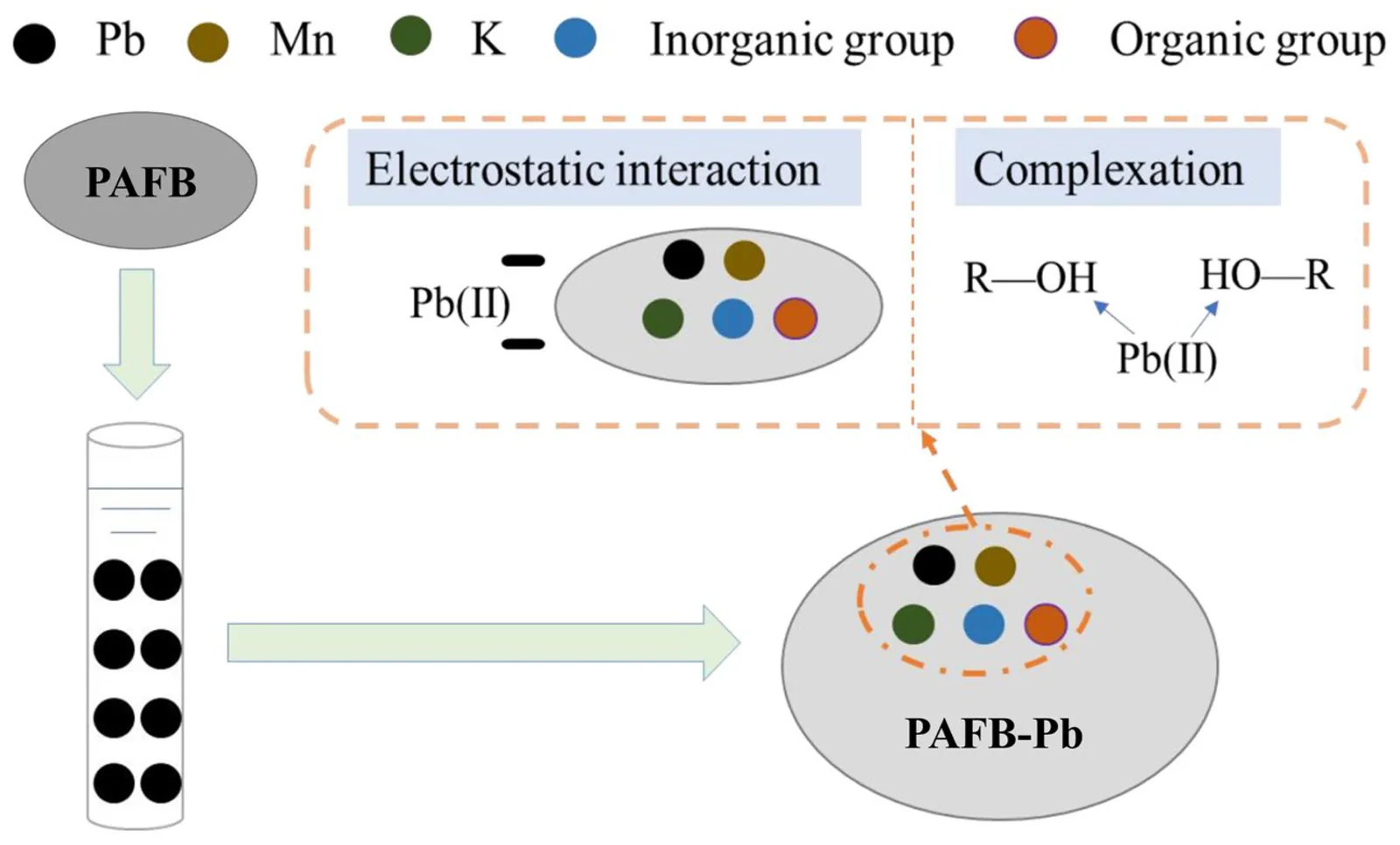
Mechanism of adsorption of Pb(II) onto PAFB.

**Table 1:** Parameters of the adsorption kinetic model.

Adsorbed Substance	Temperature	Pseudo-First-Order		
Pb(II)	25 °C	*Q*_e_ (mg/g)	*K*_1_	*R* ^2^
		7.08	18.4931	0.2953
		Pseudo-Second-Order		
		*Q*_e_ (mg/g)	*K*_2_	*R* ^2^
		9.74	0.0042	0.9997

The actual pH value of wastewater is 6.0, the initial concentration of Pb(II) is 15 mg/L, and the reaction time is 1440 min. The adsorption is carried out at an oscillation rate of 180 r/min and a temperature of 25 °C.

**Table 2:** Adsorption isothermal model parameters.

Temperature (°C)	Langmuir			Freundlich		
	*Q*_m_ (mg/g)	*K*_L_	*R* ^2^	*K*_F_	*n*	*R* ^2^
25	45.62	0.6243	0.8318	16.9631	4.7654	0.8789
35	49.62	0.5234	0.8250	17.0519	4.4006	0.9101
45	53.87	0.4491	0.8307	17.8477	4.2517	0.9363

The actual pH of wastewater is 6.0, the initial concentration of Pb(II) is 5−300 mg/L, and the reaction time is 1440 min. The adsorption is carried out at an oscillation rate of 180 r/min and temperatures of 25 °C, 35 °C, and 45 °C.

**Table 3:** Thermodynamic parameters.

Temperature (K)	Δ*G*^θ^ (kJ/mol)	Δ*S*^θ^ [J/(mol·K)]	Δ*H*^θ^ (kJ/mol)
298.15	−7.30	112.57	26.33
308.15	−8.20		
318.15	−9.56		

**Table 4:** Comparison of Pb(II) removal performance between PAFB and other similar biochar-based materials.

Adsorbent	pH	Temperature/°C	*C*_0_/(mg/L)	Isotherm Models	*Q*_m_/(mg/g)	References
PAMB	6.0	25	5–300	Langmuir	45.62	This work
Peanut shell biochar	6.0	25	60	Langmuir	42.74	[12]
Magnetic γ-Fe_2_O_3_-loaded sludge biochar.	5.0	20	10–250	Langmuir	35.27	[14]
Corn stalk biochar (550 °C)	5.0	25	10–500	Langmuir	31.30	[15]
Coffee ground biochar (800 °C)	7.0	25	10–1000	Langmuir	428.60	[16]
Pine wood biochar	5.5	25	0–400	Langmuir	20.30	[18]
Jackfruit peel biochar (400 °C)	5.7	33.85	151.30–326.2	Langmuir	83.86	[22]
Coconut shell biochar (CSBC, 500 °C)	5.0	25	20–100	Langmuir	32.31	[23]
Zn-modified CSBC (500 °C)	5.0	25	20–100	Langmuir	84.25	[23]
Mn-modified biochar	8.0	25	20–100	Langmuir	37.24	[24]

## Data Availability

The original contributions presented in this study are included in the article/Supplementary Materials. Further inquiries can be directed to the corresponding authors.

## Supplementary Materials

The following supporting information can be downloaded at: https://www.mdpi.com/article/10.3390/molecules31193437/s1, Figure S1: Isothermal adsorption/desorption curves of AF (a), AFB (b), and PAFB (c) under N_2_ atmosphere; Figure S2: The changes in the ζ potential of adsorbent with the pH values.
