# Supplementary material for: Removal of Pb(II) from Water by Potassium Permanganate-Modified Areca Fiber Biochar: Performance and Mechanism
Source: Molecules. 2026 Sep 27;31(19):3437. doi: 10.3390/molecules31193437 (PMC13665779; doi:10.3390/molecules31193437)
Supplement: Supplementary file 1 [file molecules-31-03437-s001.zip › molecules-4575659-supplementary.pdf]

Supplementary Materials

Title: Removal of Pb(II) from Water by Potassium Permanganate-Modified Areca Fiber Biochar: Performance and Mechanism

Pingping Yang [1, 2, 3], Yunquan Liu [1, 2, 3], Wanxin Cheng [1, 2, 3], Yaping Cheng [1, 2, 3, *], Wei Zhang [4*]

[1] *Guangxi Key Laboratory of Environmental Pollution Control Theory and Technology, Guilin University of Technology, Guilin, 541006, China*

[2] *University Engineering Research Center of Watershed Protection and Green Development, Guangxi, Guilin University of Technology, Guilin, 541006, China*

[3] *Key Laboratory of Carbon Emission and Pollutant Collaborative Control, Education Department of Guangxi Zhuang Autonomous Region, Guilin University of Technology, Guilin, 541006, China*

[4] *College of Civil Engineering and Architecture, China Three Gorges University, Yichang, Hubei, 443002, China*

*Corresponding author: Yaping Chen
Email: chengyaping@glut.edu.cn

**Corresponding author: Wei Zhang
E-mail: pxzwei@ctgu.edu.cn

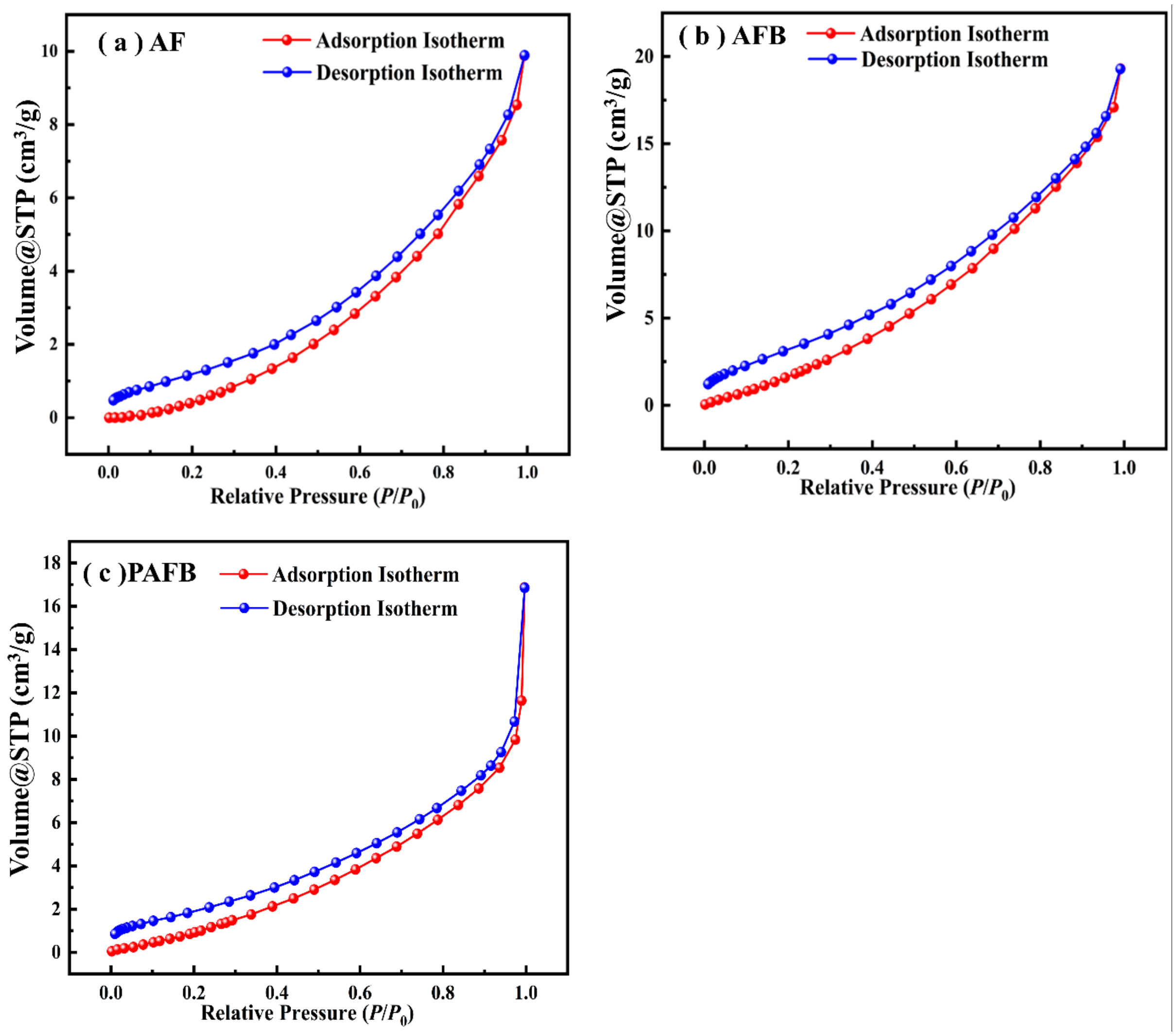

Figure S1: Isothermal adsorption/desorption curves of AF (a), AFB (b), and PAFB (c) under N_2 atmosphere

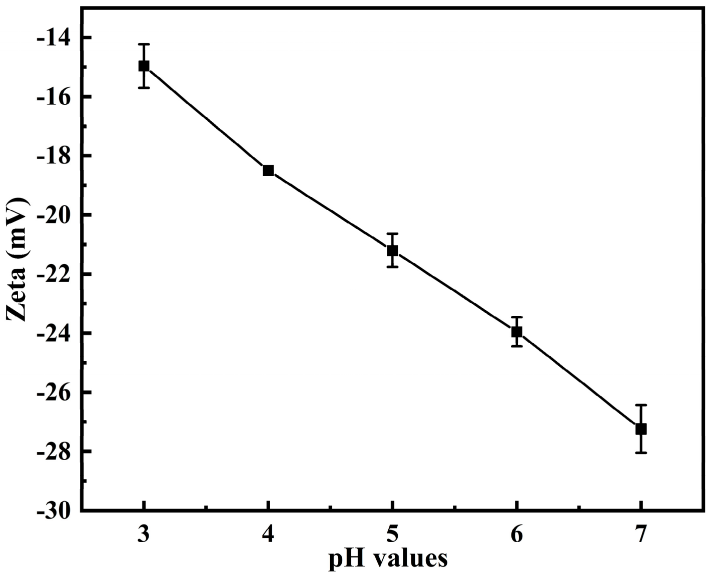

Figure S2: The changes in the ζ potential of adsorbent with the pH values
